# Loss of CXCR6 coreceptor usage characterizes pathogenic lentiviruses

**DOI:** 10.1371/journal.ppat.1007003

**Published:** 2018-04-16

**Authors:** Katherine S. Wetzel, Yanjie Yi, Anjana Yadav, Anya M. Bauer, Ezekiel A. Bello, Dino C. Romero, Frederic Bibollet-Ruche, Beatrice H. Hahn, Mirko Paiardini, Guido Silvestri, Martine Peeters, Ronald G. Collman

**Affiliations:** 1 Departments of Medicine and Microbiology, University of Pennsylvania School of Medicine, Philadelphia, PA, United States of America; 2 Division of Microbiology and Immunology, Yerkes National Primate Research Center, Atlanta, GA, United States of America; 3 UMI233-TransVIHMI/INSERM U1175, Institut de Recherche pour le Développement (IRD) and University of Montpellier, Montpellier, France; Miller School of Medicine, UNITED STATES

## Abstract

Pandemic HIV-1 originated from the cross-species transmission of SIVcpz, which infects chimpanzees, while SIVcpz itself emerged following the cross-species transmission and recombination of monkey SIVs, with *env* contributed by the SIVgsn/mus/mon lineage that infects greater spot-nosed, mustached and mona monkeys. SIVcpz and HIV-1 are pathogenic in their respective hosts, while the phenotype of their SIVgsn/mus/mon ancestors is unknown. However, two well-studied SIV infected natural hosts, sooty mangabeys (SMs) and African green monkeys (AGMs), typically remain healthy despite high viral loads; these species express low levels of the canonical coreceptor CCR5, and recent work shows that CXCR6 is a major coreceptor for SIV in these hosts. It is not known what coreceptors were used by the precursors of SIVcpz, whether coreceptor use changed during emergence of the SIVcpz/HIV-1 lineage, and what T cell subsets express CXCR6 in natural hosts. Using species-matched coreceptors and CD4, we show here that SIVcpz uses only CCR5 for entry and, like HIV-1, cannot use CXCR6. In contrast, SIVmus efficiently uses both CXCR6 and CCR5. Coreceptor selectivity was determined by Env, with CXCR6 use abrogated by Pro326 in the V3 crown, which is absent in monkey SIVs but highly conserved in SIVcpz/HIV-1. To characterize which cells express CXCR6, we generated a novel antibody that recognizes CXCR6 of multiple primate species. Testing lymphocytes from SM, the best-studied natural host, we found that CXCR6 is restricted to CD4+ effector memory cells, and is expressed by a sub-population distinct from those expressing CCR5. Thus, efficient CXCR6 use, previously identified in SM and AGM infection, also characterizes a member of the SIV lineage that gave rise to SIVcpz/HIV-1. Loss of CXCR6 usage by SIVcpz may have altered its cell tropism, shifting virus from CXCR6-expressing cells that may support replication without disrupting immune function or homeostasis, towards CCR5-expressing cells with pathogenic consequences.

## Author summary

Use of an entry coreceptor, in conjunction with CD4, is the main determinant of HIV/SIV cell targeting, which in turn may be a central factor determining pathogenicity of infection. Two SIVs that generally do not cause AIDS in their natural hosts, SIVsmm of sooty mangabeys and SIVagm of African green monkeys, express low levels of the canonical coreceptor CCR5 and have been shown recently to enter CD4+ T cells via CXCR6 in addition to CCR5. Here we asked which entry coreceptors are used by SIVcpz, the HIV-1 precursor that is pathogenic in chimpanzees, and by SIVmus, a member of the monkey SIV lineage that contributed the *env* gene of SIVcpz. We found that SIVcpz uses only CCR5, while SIVmus efficiently uses both CXCR6 and CCR5 for entry. We then examined CXCR6 expression on sooty mangabey CD4+ T cells, and found it is present on a subset of differentiated memory T cells distinct from cells expressing CCR5. Coreceptor-mediated cell targeting thus differs among primate lentiviruses. Entry via CXCR6 in some primate hosts may target SIV towards cells that may support viremia without causing immunodeficiency, but this pattern was lost during the emergence of pathogenic SIVcpz and HIV-1 infections.

## Introduction

Pandemic HIV-1 resulted from the cross-species transmission of SIVcpz, which infects chimpanzees (*Pan troglodytes*), into humans [1, 2]. SIVcpz itself emerged in chimpanzees following the cross-species transmission and recombination of SIVs infecting monkeys on which chimpanzees prey [3, 4]. The 5’ half of SIVcpz, which encodes *gag* and *pol*, is derived mainly from SIVrcm that infects red-capped mangabeys (RCM, *Cercocebus torquatus*) [3, 5]. The 3’ half of SIVcpz, which encodes *env*, originated from a member of the SIVgsn/mus/mon lineage, which infects greater spot-nosed monkeys (GSN, *Cercopithecus nictitans*), mustached monkeys (MUS, *Cercopithecus cephus*) and mona monkeys (MON, *Cercopithecus mona*), respectively [6, 7]. RCMs, GSNs, MUSs and MONs are among more than forty African primate species known to be naturally infected with species-specific strains of SIV [8]. While the outcome of infections of those four hosts have not been characterized, certain other naturally infected monkeys, namely sooty mangabeys (SM, *Cercocebus atys*) and African green monkeys (AGM, *Chlorocebus spp*.), do not typically progress to AIDS [8, 9]. Similarly, SIVmac infection of rhesus (RM, Macaca mulatta) and other macaque species, which serves as the primary animal model for AIDS, is the result of cross-species transmission of SIVsmm from SM. The lack of pathogenicity in SMs and AGMs is believed to be the product of millions of years of virus-host coevolution, while the more recent introduction of HIV-1, SIVcpz and SIVmac into a “non-natural” host is believed to underlie, at least in part, the pathogenic outcomes of these infections [10].

Like HIV-1/human and SIVmac/RM infections, infected SMs and AGMs exhibit high viral loads and rapid turnover of infected cells [11–14], indicating that lack of disease progression in these species is not due to viral control or absence of cytopathogenicity. Nevertheless, these natural hosts lack key features of pathogenic infection such as progressive CD4+ T cell loss, lymph node inflammation and fibrosis, gut barrier breakdown and microbial translocation, and chronic immune activation [15]. Instead, CD4+ T cells in the blood are generally preserved, CD4+ T cell loss in the gut is less extensive and recovers, lymph node structure and function are maintained, gut integrity is preserved, microbial translocation is absent in chronic infection, and generalized immune activation resolves after acute infection [9, 16]. Lack of disease in SMs and AGMs has been ascribed to limited infection and/or loss of specific CD4+ T cell types that are prominent targets in HIV-1 infection, such as central memory (Tcm) and stem-cell memory (Tscm) subsets [17, 18], Th17 cells [19–21], and T follicular helper (Tfh) cells in lymphoid tissue [22]. These observations raise the question whether cell targeting has changed during the emergence of the SIVcpz/HIV-1 lineage.

HIV and SIV target cells are largely defined by expression of CD4 plus a 7 transmembrane G protein-coupled receptor (7TMR) that serves as a coreceptor. HIV-1 infection requires the chemokine receptor CCR5 [23–25], although acquisition of CXCR4 use is sometimes seen late in infection [26, 27]. SIVsmm, SIVagm and other SIVs that have been studied typically use CCR5 for entry, but very rarely use CXCR4 [28–31]. It has long been known that SIVs can enter cells *in vitro* using additional 7TMRs, including CXCR6 and GPR15 (usually tested with molecules of human origin) [32–34], but CCR5 was thought to be the sole coreceptor responsible for SIV entry and thus cell targeting *in vivo*. A key observation made several years ago was that CCR5 expression was exceedingly low on primary CD4+ T cells of four natural hosts, including AGMs and SMs, in contrast to humans and RMs that express robust levels of this coreceptor [35]. It was thus postulated that restricted expression of CCR5 would protect critical cells from infection [17, 18, 35]. However, it remained unclear how high levels of viremia could be supported by such low CCR5 expressing cells and whether virus was specifically targeting CD4+ T cells capable of supporting robust viral replication without causing immune disruption.

In contrast to the general dogma of SIV CCR5 dependence, it was discovered early on that RCMs frequently lacked functional CCR5 due to a common deletion mutation, and that SIVrcm used human CXCR6 and CCR2b but not CCR5 to enter transfected cells [36, 37]. More recently, we discovered that SMs also harbor a common CCR5 deletion, and that SMs lacking functional CCR5 were infected with SIVsmm at the same frequency as wild-type animals and maintained high viral loads [38]. We then identified species-matched CXCR6 as an efficient SIVsmm entry pathway *in vitro* and in primary SM lymphocytes *ex vivo* [39, 40]. We also showed that CXCR6 is a robust coreceptor *in vitro* and in primary cells *ex vivo* for SIVagmSab that infects sabaeus AGMs [41]. SIVagmVer, which infects vervet AGMs, has also been shown to not require CCR5 for primary cell infection *ex vivo*, and to use CXCR6 efficiently *in vitro* [42]. Thus, efficient use of CXCR6, instead of or in addition to CCR5, appears to be a common feature of virus/host interactions in multiple natural host infections, where CCR5 expression is low or, in some cases, genetically absent. This has raised key questions: (a) what entry pathways are used by the HIV-1 precursor SIVcpz, and its upstream guenon SIV forerunner that supplied its *env* gene; (b) was there a change in coreceptor use that occurred during the emergence of SIVcpz and HIV-1; and (c) what cells express CXCR6 in natural host species known to harbor CXCR6-using SIV, and are therefore targets for infection?

In this study we show that SIVcpz exhibits a pattern of coreceptor use similar to HIV-1, in that it is restricted to CCR5 and does not use species-matched CXCR6. In contrast, SIVmus, whose ancestors contributed the *env* gene of SIVcpz, efficiently uses species-matched CXCR6 in addition to CCR5. Importantly, this more restricted coreceptor tropism of SIVcpz compared to SIVmus is determined by its viral Env, rather than the host-specific coreceptor sequence, with residue 326 in the V3 crown of Env playing a major role. We also developed a novel antibody that recognizes CXCR6 from multiple nonhuman primate species, and show that in SMs, CXCR6 is restricted to the effector memory subset of CD4+ T cells, and is expressed on a cell population distinct from that expressing CCR5. These findings support a model where CXCR6 use is a characteristic feature of natural monkey SIV infections, including the lineage from which SIVcpz/HIV-1 emerged, and that the loss of this coreceptor usage is associated with altered CD4+ T cell targeting to subpopulations that may be less able to tolerate SIVcpz/HIV-1 infection.

## Results

### Cloning and analysis of representative coreceptor and CD4 genes from chimpanzees, mustached monkeys and greater spot-nosed monkeys

We first aimed to define the coreceptor use patterns of SIVcpz and members of the SIVgsn/mus/mon lineage. Given that single amino acid differences between species can alter coreceptor function [43], it is important to test each virus on cells expressing species-matched coreceptor and CD4 molecules. We focused our analysis on CXCR6 because it mediates primary lymphocyte infection in two natural host species, SMs [40] and sabaeus AGMs [41]. We also examined GPR15, which is used by SIVagm and SIVsmm *in vitro* [39, 41], CXCR4, which is used by certain HIV-1 isolates but not SIVs, and CCR5.

Using stored chimpanzee (*P*. *t*. *verus*) peripheral blood mononuclear cells (PBMC), we amplified and cloned cpzCXCR6, cpzGPR15 and cpzCCR5 from genomic (g)DNA, and cpzCXCR4 from cDNA. Because central (*P*. *t*. *troglodytes*) and eastern (*P*. *t*. *schweinfurthii*,), but not western (*P*. *t*. *verus*) chimpanzees harbor SIVcpz in the wild [2, 44, 45], we first asked whether the cloned *P*. *t*. *verus* coreceptor sequences differed from those of other chimpanzee subspecies. The *P*. *t*. *verus* coreceptor sequences were aligned with the corresponding genes from four *P*. *t*. *troglodytes* and six *P*. *t*. *schweinfurthii* chimpanzees [46], as well as to published *P*. *t*. *troglodytes* CCR5 and CXCR4 alleles [47]. Our *P*. *t*. *verus* CXCR6, CXCR4 and CCR5 clones were identical to both *P*. *t*. *troglodytes* and *P*. *t*. *schweinfurthii* sequences at the amino acid level, although several synonymous SNPs were seen. The *P*. *t*. *troglodyte*s deep sequence data contained two GPR15 alleles, encoding either Ser276 or Pro276 within the predicted third extracellular loop (ECL3), while the *P*. *t*. *schweinfurthii* deep sequence data were identical to the Pro276 allele. The *P*. *t*. *verus* cpzGPR15 allele cloned here matched the Pro276 sequence. Also, the CXCR6 and GPR15 amino acid sequences cloned here match previously published alleles from captive chimpanzees of an unspecified subspecies [48]. Thus, the coreceptors derived here from *P*. *t*. *verus* are suitable for investigating SIVcpz entry.

The 3’ half of SIVcpz, which encodes Env, derives from an ancient SIV of the SIVgsn/mus/mon lineage. In order to best approximate coreceptor use of that SIVcpz Env ancestor, we tested species-matched coreceptor usage of present day virus of this lineage. We were unable to access PBMC or other viable cells from these species, but did obtain gDNA from MUS and GSN bushmeat [6, 7, 49, 50]. CCR5, CXCR6 and GPR15, which are each encoded by a single exon, were cloned from MUS and GSN gDNA using primers flanking the ORF. To clone CXCR4, the two exons were amplified individually and spliced together to generate the full coding sequence.

**Fig 1:**
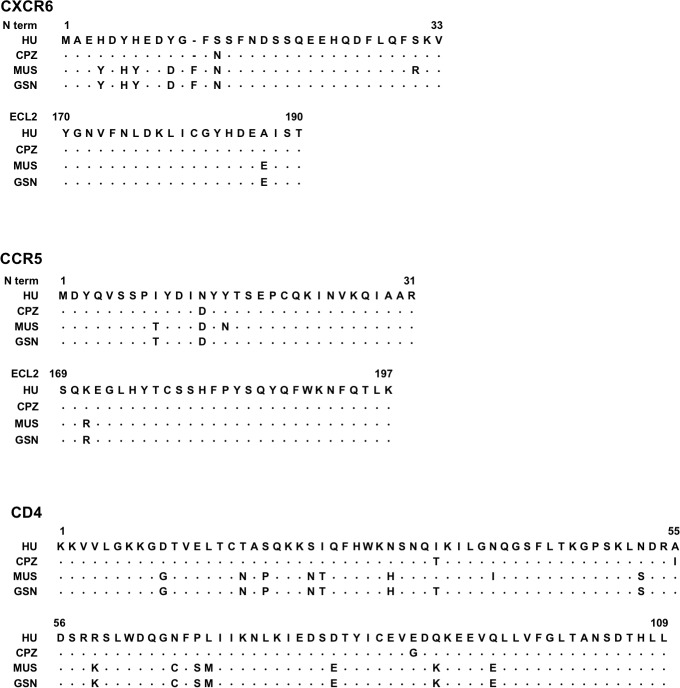
Coreceptor and CD4 sequences vary in sequence between human, CPZ, GSN and MUS. CXCR6 and CCR5 were cloned from CPZ, GSN and MUS genomic DNA. Sequences were aligned to the human sequence, and shown are the N-terminus (N term) and the second extracellular loop (ECL2), the two domains thought to interact with the virus glycoprotein, based on studies of human CCR5 and HIV-1 gp120. GSN and MUS CD4 exons were sequenced from genomic DNA and aligned to known sequences of human CD4 and chimpanzee CD4; shown is domain 1 that interacts with HIV/SIV gp120. Residues identical to the human sequence are represented as dots.

One musCCR5 allele has been previously described [51] and the allele cloned here differed at one amino acid, encoding Pro at position 35 instead of Leu. GsnCCR5 has been reported to be polymorphic [51], and the molecule cloned here matched a previously described allele at the amino acid level. No prior sequences are available for CXCR6, GPR15 or CXCR4 of either species. Fig 1 shows amino acid sequences of CXCR6 and CCR5 of MUS and GSN aligned to those of chimpanzee and human within the N-terminus (N-term) and second extracellular loop (ECL2) regions, the domains likely involved in coreceptor-Env interactions based on studies of HIV-1 and human coreceptors [52]. The N-termini of musCXCR6 and gsnCXCR6 are quite distinct from the human and chimpanzee molecules, differing by at least five residues. In contrast, the N-termini of musCCR5 and gsnCCR5 are quite similar to those of humans and chimpanzee, except for the absence in musCCR5 of one of the four Tyr in this region (Tyr15) that in human CCR5 are known to be sulfated and important for Env binding and coreceptor function [53]. Interestingly, we found that musCXCR6 encoded Arg at position 31, which is present in CXCR6 of macaques and renders rmCXCR6 a poor coreceptor for SIVmac [43]. In contrast, gsnCXCR6 encoded the more common Ser31 that is associated with robust CXCR6 coreceptor function.

We then generated musCD4 and gsnCD4 clones. Since cDNA was not available, we sequenced the nine CD4 exons from gDNA and synthesized full-length clones based on those sequences. Within Domain 1, which interacts with the SIV/HIV Env, the two species were similar but not identical (Fig 1). However, they both differed from human CD4 at 16 out of 109 residues in this region, and from the chimpanzee CD4 allele used here (which had been previously cloned [54]) at 19 (MUS) or 17 (GSN) residues.

### SIVcpz is restricted to cpzCCR5 for entry but SIVmus uses musCXCR6

**Fig 2:**
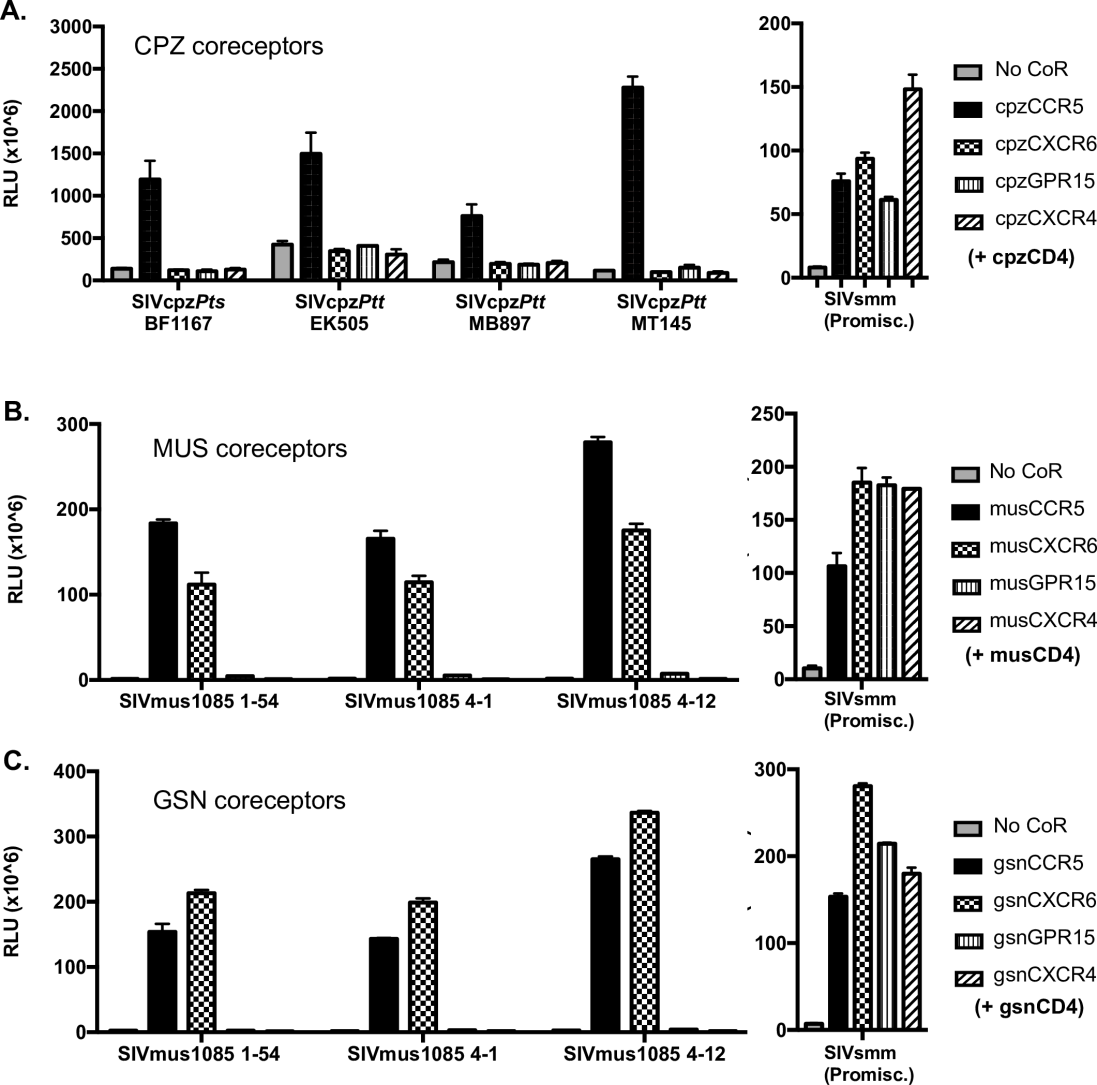
SIVcpz is restricted to CPZ CCR5, while SIVmus1085 can use MUS and GSN CXCR6 and CCR5 for entry. A) Left panel: CF2thLuc cells that contain a Tat-driven luciferase reporter were transfected with expression plasmids containing chimpanzee coreceptor and CD4 or empty vector (No CoR). 48 hours later cells were infected with one of four diverse SIVcpz isolates derived from infectious molecular clones. Entry was quantified 48 hours later by lysing cells and measuring luciferase content by relative light units (RLU). Right panel: 293T cells were transfected with cpzCD4 and coreceptor and infected with luciferase reporter pseudotypes expressing a promiscuous SIVsmm Env that can use a wide repertoire of coreceptors for entry. B-C) 293T cells were transfected with expression plasmids containing coreceptor or empty vector (No CoR), in conjunction with CD4, of MUS (B) or GSN (C) origin. 48 hours later cells were infected with luciferase reporter viruses carrying SIVmus1085 Envs (left panel) or a promiscuous SIVsmm (right panel). Entry was quantified 72 hours later by lysing cells and measuring luciferase content by relative light units (RLU). Infections were carried out in triplicate and data represent means ± one standard deviation.

To test coreceptor usage of SIVcpz, expression vectors containing each CPZ candidate coreceptor and cpzCD4 were transfected into CF2th-Luc cells that contain a Tat-driven luciferase reporter [55]. Cells were then infected with four SIVcpz strains reflecting diverse host and molecular lineages [56]: SIVcpz*Pts* BF1167, isolated from *P*. *t*. *schweinfurthii*, and SIVcpz*Ptt* EK505, MB897, and MT145, all isolated from *P*. *t*. *troglodytes*. For all SIVcpz isolates tested, only cpzCCR5 supported virus entry (Fig 2A, left panel). Since no entry occurred through cpzCXCR6, cpzCXCR4 or cpzGPR15, we confirmed that these molecules would be expressed and functional by testing a pseudotyped SIVsmm variant that was previously shown to use a wide range of human, SM, AGM and macaque coreceptors [39]. All three CPZ coreceptors enabled entry by this promiscuous variant (Fig 2A, right panel). We also tested SIVcpz use of additional 7TMRs of chimpanzee origin, APJ, CCR2b, CCR3, CCR4, CCR8, and GPR1, and found that none of these were functional coreceptors for any of the four strains either (S1 Fig). Therefore, SIVcpz is restricted to the use of CCR5 as a coreceptor and, like HIV-1 but unlike SIVsmm and SIVagm, does not use species-matched CXCR6 or GPR15.

Because no functional envelopes or infectious molecular clones of the SIVgsn/mus/mon lineage have been reported, we first needed to amplify functional *envs*. We successfully amplified full-length *envs* from gDNA from bushmeat of two SIV-infected MUS samples (1085 and 1246), cloned them into expression vectors, and screened for function by generating pseudotyped viruses and testing entry via CCR5. Several *envs* cloned from MUS1085 mediated robust infection, while those from MUS1246 functioned only weakly. We therefore tested coreceptor use with the 3 *envs* amplified from MUS1085 (1–54, 4–1 and 4–12). These Envs showed 96 to 98% amino acid identity to each other, and to the previously reported Env sequence from this animal [6].

To test SIVmus coreceptor use, luciferase reporter pseudotypes were generated carrying the SIVmus1085 Envs and used to infect 293T cells transfected with musCD4 and MUS coreceptors, or gsnCD4 and GSN coreceptors (Fig 2B and 2C, left panels). In contrast to SIVcpz, SIVmus entered cells expressing species-matched CXCR6 as well as CCR5. SIVmus also used CXCR6 and CCR5 of the closely related GSN species. In contrast, SIVmus did not use GPR15 from either species, even though the GPR15 molecules were functional for entry by the promiscuous SIVsmm strain (Fig 2B and 2C, right panels). Interestingly, the Arg31 residue present in musCXCR6 did not prevent entry through this coreceptor, even though it is present in rhesus macaque CXCR6 and is responsible for restricted entry of SIVmac through rmCXCR6 [43].

Therefore, SIVmus enters cells expressing CXCR6 and CCR5, like SIVsmm and SIVagm, while SIVcpz only enters cells expressing CCR5, like HIV-1.

### Restricted use of species-matched CXCR6 by SIVcpz is a property of the virus, not the coreceptor

**Fig 3:**
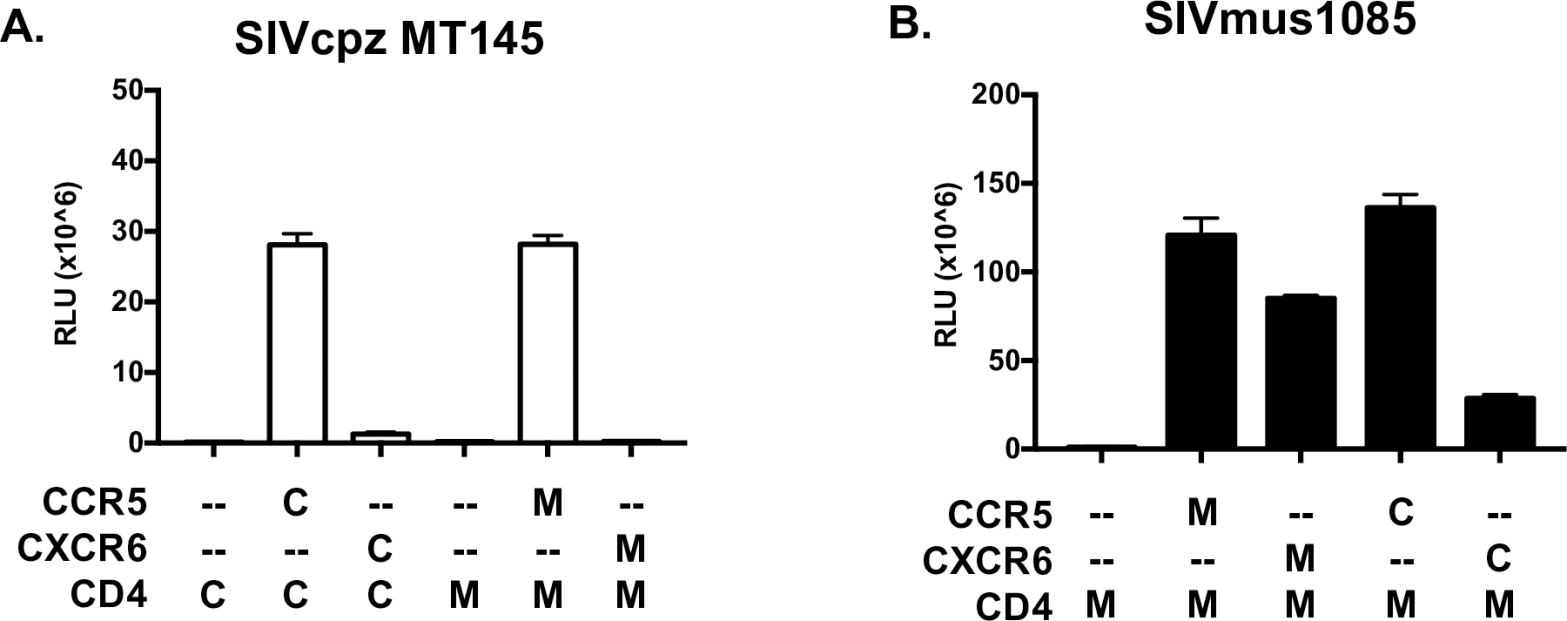
The inability of SIVcpz to use CXCR6 for entry is determined by Env, not cpzCXCR6. 293T cells were transfected with expression plasmids containing CD4 and coreceptor. The species origin of the CD4 and coreceptor are indicated below the graph (C, chimpanzee; M, mustached monkey;–, empty vector control). 48 hours post transfection, cells were infected with luciferase reporter pseudotypes carrying the SIVcpz MT145 Env (A) or the SIVmus1085 1–54 Env (B). Entry was quantified 72 hours later by lysing cells and measuring luciferase content by relative light units (RLU). Infections were carried out in triplicate and data represent means ± one standard deviation.

The inability of SIVcpz to use cpzCXCR6 could be due to either the SIVcpz Env, or the species origin and sequence of the coreceptor. Therefore, we first probed coreceptor origin by testing SIVcpz entry into cells expressing musCXCR6 and musCD4 (Fig 3A). The SIVcpz MT145 pseudotype entered cells expressing musCCR5 and musCD4, but not those expressing musCXCR6 and musCD4 (Fig 3A). Identical results were seen with SIVcpz EK505 (S2A Fig).

We then asked whether cpzCXCR6 could function as a coreceptor for SIVmus. Pseudotyped virus carrying the SIVmus1085 1–54 Env was used to infect cells expressing musCD4 with or without musCCR5, musCXCR6, cpzCCR5 or cpzCXCR6. SIVmus1085 entered through each of these coreceptors (Fig 3B), although entry through cpzCXCR6 was somewhat less robust. Identical results were seen with SIVmus1085 4–12 (S2B Fig). As shown in Fig 2A, cpzCXCR6 also supports entry of the promiscuous SIVsmm isolate. Together these results indicate that the inability of SIVcpz to enter through CXCR6 is a property of the SIVcpz Env, rather than an intrinsic defect in coreceptor function by cpzCXCR6.

### The SIVcpz V3 loop Pro326 sequence restricts CXCR6 use

**Fig 4:**
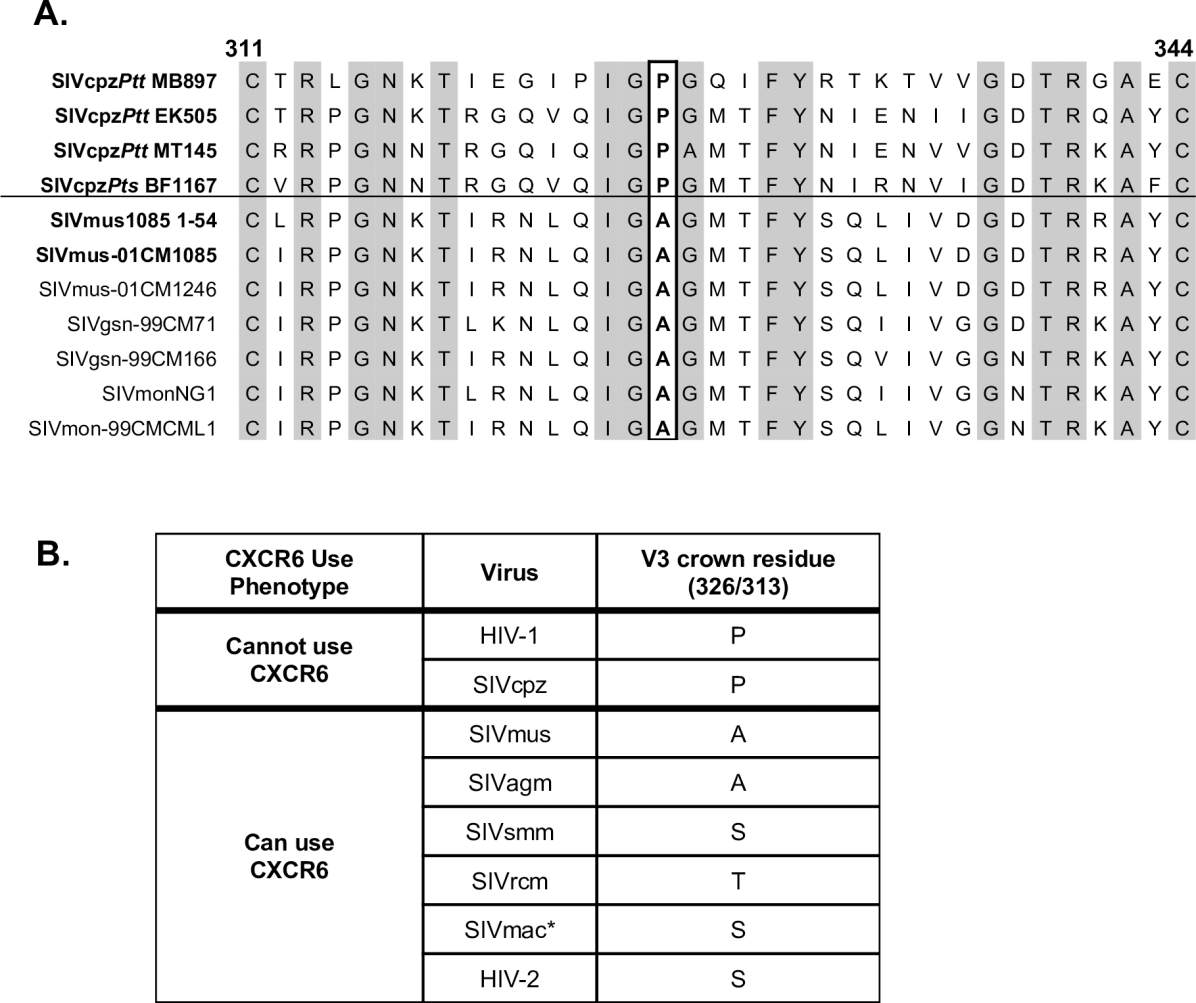
Amino acid sequences of SIV and HIV V3 loops. A) The V3 loop amino acid sequences of SIVcpz and SIVmus sequences used in this study (bold text) were aligned with other members of the SIVgsn/mus/mon lineage (plain text) using the ClustalW algorithm. The V3 loop of the previously published SIVmus-01CM1085 that was isolated from the same animal as the SIVmus1085 *envs* cloned here is identical to that of SIVmus1085 4–1 and 4–12. Residue 326 (SIVmac239 numbering) of the V3 crown is boxed. Conserved residues are shaded in grey. B) The predominant amino acid residues that occur at V3 crown residue 326 (SIVmac239 numbering) or 313 (HIV HXB2 numbering) for SIVs and HIVs where use of CXCR6 has been tested. *SIVmac can use other species’ CXCR6 for entry, but does not efficiently enter through rmCXCR6 due to a S31R polymorphism in the N terminus of rmCXCR6.

The distinct capacities of SIVcpz and SIVmus to enter through CXCR6 provided an opportunity to probe Env determinants of CXCR6 usage. Since the V3 loop of Env is often a principal determinant of coreceptor use (both in HIV-1 and SIV [57, 58]), we aligned this region of the Envs tested in this study (Fig 4A, bold text), as well as additional published sequences in the SIVgsn/mus/mon lineage (Fig 4A, plain text). The SIVcpz strains each had Pro in the crown of the V3 loop at residue 326 (SIVmac239 numbering), but members of the SIVgsn/mus/mon lineage had Ala in this position. We then compared this residue across SIV and HIV strains for which the ability to use CXCR6 has been tested and reported. HIV-1, in which CXCR6-using strains are very rare, almost invariably encodes Pro, like SIVcpz. (Pro is present at this position in 100% of SIVcpz and 95% of HIV-1 sequences in the Los Alamos National Laboratory HIV Sequence database.) In contrast, Pro is absent in widely divergent SIV or HIV-2 strains that can use CXCR6, which instead encode at that position Ala (SIVmus, SIVagm), Thr (SIVrcm) or Ser (SIVsmm, SIVmac, HIV-2) [59].

**Fig 5:**
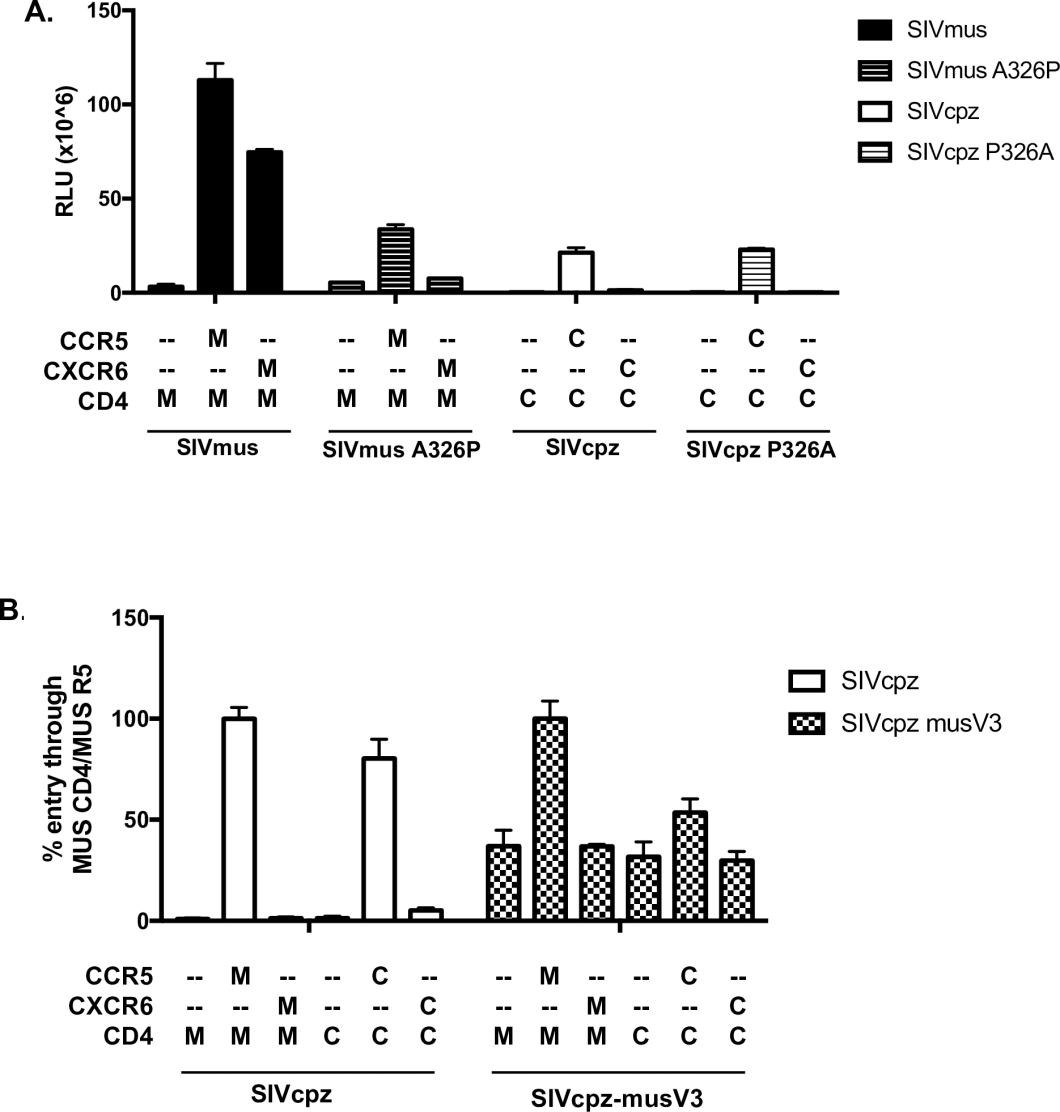
SIVmus1085 Env determinants of CXCR6 usage. A) SIVmus1085 1–54 and SIVcpz MT145 *env* constructs were mutated to generate V3 loop mutants SIVmusA326P and SIVcpzP326A. Luciferase reporter pseudotyped viruses carrying these Envs, or SIVmus 1085 1–54 or SIVcpz MT145, were used to infect 293T cells expressing musCD4 and coreceptor (M) or cpzCD4 and coreceptor (C). Entry was quantified 72 hours post-infection by relative light units (RLU). B) The V3 loop of SIVcpz MT145 was replaced with the V3 loop of SIVmus1085 1–54 to generate SIVcpz-musV3. Pseudotypes carrying the SIVcpz-musV3 Env or SIVcpz MT145, were used to infect 293T cells expressing musCD4 and coreceptor (M) or cpzCD4 and coreceptor (C). Due to low infectivity of SIVcpz-musV3, entry was normalized to % entry through musCD4 and musCCR5 to enable comparison. Infections were carried out in triplicate and data represent means ± one standard deviation.

Based on this association, we tested whether Pro326 might regulate utilization of CXCR6. Site-directed mutagenesis was used to generate SIVmusA326P, which encodes Pro instead of the native Ala at position 326, and SIVcpzP326A, which encodes Ala instead of Pro. As shown in Fig 5A, introduction of Pro into the SIVmus V3 crown (A326P) completely abrogated its ability to enter cells through musCXCR6, despite retaining the capacity to use musCCR5. However, introduction of Ala into the SIVcpz V3 crown (P326A) mutant did not confer the ability to use musCXCR6. Thus, Pro326 as found in SIVcpz is incompatible with CXCR6 usage, although Ala as found in SIVmus is not sufficient to enable CXCR6 use.

We then asked if the entire SIVmus V3 loop was sufficient to confer CXCR6 use to SIVcpz by introducing the V3 loop of SIVmus into the SIVcpz Env (Fig 5B). The resulting virus was poorly infectious but, nevertheless, did not suggest rescue of either musCXCR6 or cpzCXCR6 usage. Thus, the SIVcpz Pro326 restricts CXCR6 use, but Env domains beyond the SIVmus V3 loop likely contribute to its efficient use of CXCR6.

### CXCR6 is expressed on natural host CD4+ lymphocytes and defines a population distinct from CD4+ CCR5+ T cells

**Fig 6:**
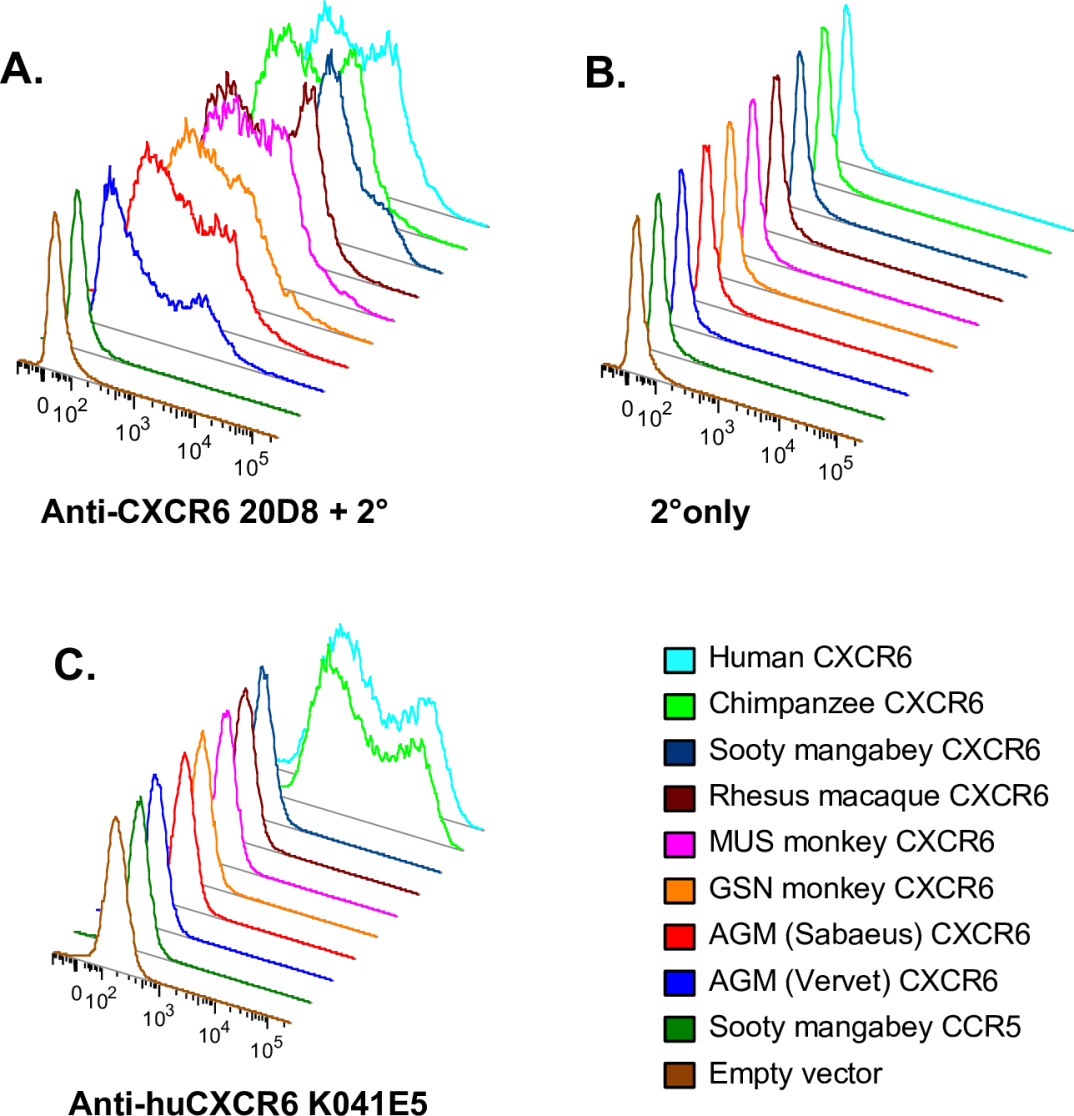
Monoclonal antibody 20D8 recognizes multiple primate species’ CXCR6 molecules. 293T cells were transfected with expression plasmids containing one of eight primate CXCR6 genes, SM CCR5 or empty vector. 48 hours later, cells were stained with mouse anti-primate CXCR6 antibody 20D8 followed by a goat anti-mouse secondary (A), secondary only (B) or a commercially available anti-human CXCR6 antibody (C).

Given the use of CXCR6 by SIVagm, SIVsmm and now SIVmus, we asked what CD4+ cells in natural hosts express CXCR6, thus potentially serving as targets for SIV. Studies of non-human primate CXCR6 expression have been hindered by the fact that available anti-human CXCR6 antibodies do not recognize CXCR6 molecules of monkeys (Fig 6C and [60]). Therefore, we generated an anti-CXCR6 antibody by immunizing mice with three doses of plasmid DNA encoding smCXCR6 followed by five intraperitoneal injections of murine B78H1 cells transduced to express smCXCR6. A cell-based ELISA was used to screen hybridoma supernatants for detection of CXCR6, yielding antibody clone 20D8. Transfection of 293T cells with plasmids to express various coreceptor molecules followed by flow cytometry showed that 20D8 recognizes CXCR6 from all primates tested, including SM, RM, AGM, MUS, GSN and both human and chimpanzee, whereas current commercially available antibodies recognize only human and chimpanzee (Fig 6A–6C). mAb 20D8 is specific for CXCR6, as it does not react with sooty mangabey CCR5, CXCR4, GPR1 or APJ (S3A Fig). Furthermore, sorting of PBMC based on staining by this antibody results in a population enriched for CXCR6 mRNA (S3B Fig).

**Fig 7:**
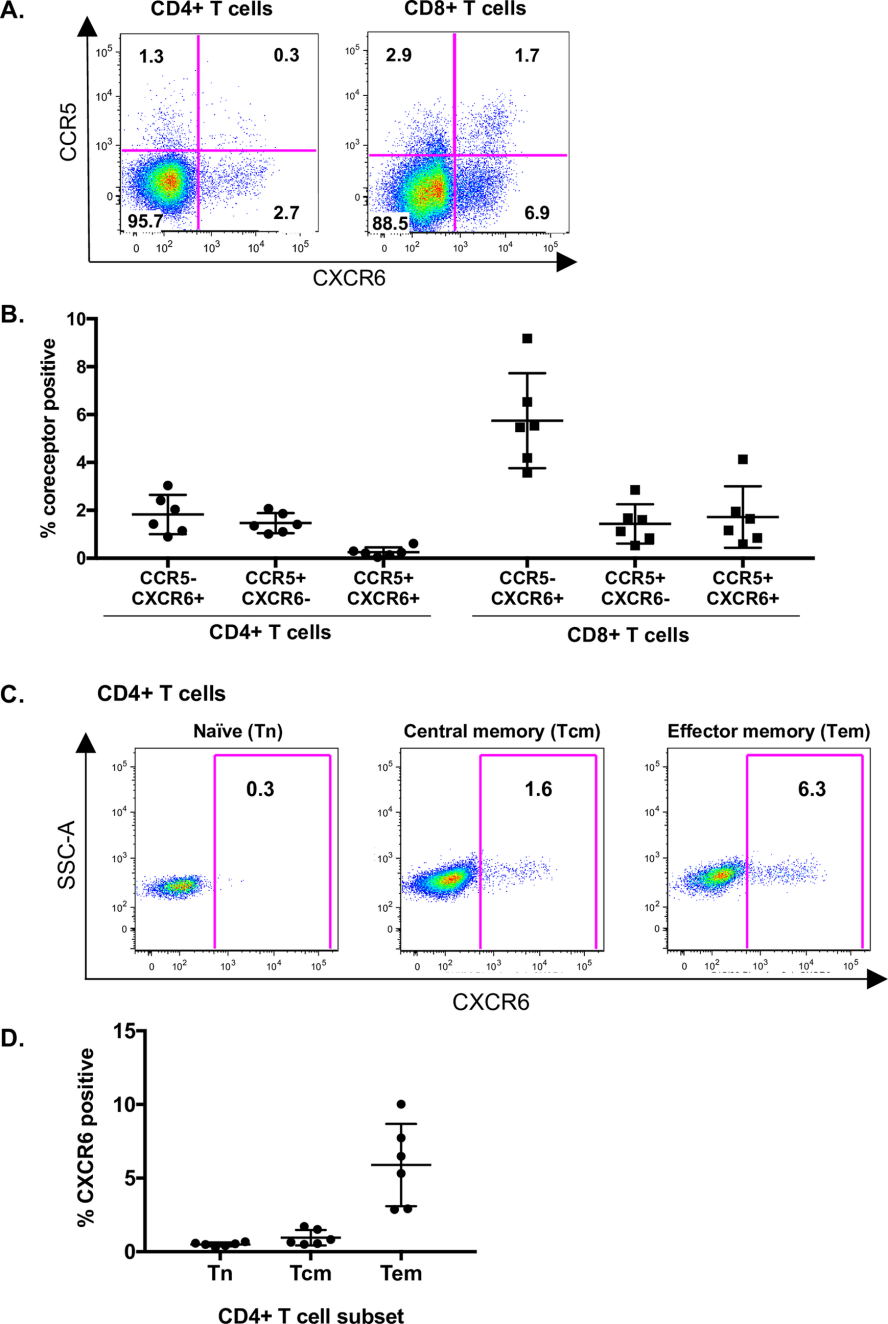
CXCR6 is expressed on natural host CD4+ T cells that are distinct from CCR5-expressing cells. A) CCR5 (y-axis) and CXCR6 (x-axis) expression on resting SM CD4+ T cells (left) and CD8+ T cells (right). Numbers in the quadrant are the percent of CD4+ or CD8+ T cells expressing the respective combination of coreceptors. One representative animal is shown. B) Summary of CCR5 and CXCR6 single- and co-expressing cells as the percent of total CD4+ and CD8+ T cells from six SMs. Each symbol represents cells from a different individual SM. C) Resting SM PBMC were stained using antibodies to define CXCR6 expression of CD4+ memory subsets: naive (Tn: CD45RA+/ CCR7+/ CD28+/ CD95-), central memory (Tcm: CD45RA-/ CCR7+) and effector memory (Tem: CD45RA-/ CCR7-). D) Summary of CXCR6 expression of CD4+ Tn, Tcm and Tem from six SMs. Each symbol represents cells from a different individual SM. Data show individual percentages, along with mean and standard deviation.

To examine CXCR6 expression in an SIV natural host, we used primary lymphocytes from SMs. SMs are known to support robust SIVsmm infection *in vivo* through non-CCR5 pathways [38], and CXCR6 mediates entry into primary SM lymphocytes *ex vivo* [40]. We first used mAb 20D8 to define CXCR6 expression on resting SM PBMC. This showed that CXCR6 is expressed on both CD4+ and CD8+ T cells, with a greater proportion of CD8+ cells positive for CXCR6 (Fig 7A). Notably, CXCR6 and CCR5 were expressed on largely distinct, non-overlapping populations in both CD4+ and CD8+ T cells (Fig 7A and 7B). We quantified expression on PBMC from six SMs (Fig 7B), and found that CXCR6+CCR5- cells represented an average of 2% of SM CD4+ T cells, while 1.5% of CD4+ T cells were CXCR6-CCR5+, and only 0.3% of CD4+ T cells were dual positive CCR5+CXCR6+ (Fig 7B). A larger proportion of CD8+ T cells expressed CXCR6, with the majority being CXCR6+CCR5- (Fig 7A and 7B).

We then examined the memory subsets of CD4+ T cells that express CXCR6 (Fig 7C and 7D). CXCR6 expression was enriched on effector memory CD4+ T cells (Tem; 5.9% of CD45RA-/CCR7- CD4+ T cells), but infrequent on naïve CD4+ T cells (Tn; 0.5% of CD45RA+/CCR7+/CD28+/CD95- CD4+ T cells) and central memory CD4+ T cells (Tcm; 1.0% of CD45RA-/CCR7+ CD4+ T cells) (Fig 7D). We also examined CXCR6 expression on RM PBMC (although macaque CXCR6 is not an efficient coreceptor for SIVmac [40, 42, 43]). Like SM, CXCR6 expression in RM was enriched on CD4+ Tem cells over Tn and Tcm, and defined a population of cells distinct from those expressing CCR5 (S4A Fig). Thus, the pattern of CXCR6 expression is similar in SM and RM CD4+ T cells.

We then asked whether CXCR6 expression of SM PBMC changed upon stimulation (S5 Fig). Following stimulation with concanavalin A, the proportion of CD4+ T cells expressing CXCR6 increased from 1.6% to 5.6%, while the proportion of cells expressing CCR5 did not increase above baseline as has been reported previously [17]. Five days post stimulation, the proportion of CD4+ T cells expressing CXCR6 (5.6%) exceeded that of CD4+ T cells expressing CCR5 (1.2%). Thus, CXCR6 expression is upregulated on CD4+ T cells in response to mitogen stimulation, and to an extent greater than CCR5, indicating distinct regulation patterns.

Thus, in natural host SMs, CXCR6 is expressed on differentiated Tem CD4+ T cells, and is on a population distinct from those expressing CCR5. This finding suggests that SIV entry via CXCR6 will result in a unique distribution of target cells different from those targeted by virus in non-natural hosts such as humans, which express abundant CCR5 and whose viruses do not use CXCR6.

## Discussion

Use of CXCR6 for entry is a shared feature of the natural host viruses SIVsmm and SIVagm. Here we identified SIVmus as yet another SIV that can robustly enter cells expressing species-matched CXCR6, extending this observation to the SIV lineage that gave rise to SIVcpz/HIV-1. In contrast, SIVcpz, which is pathogenic in its chimpanzee host, is restricted to use of CCR5, like HIV-1. This more restricted coreceptor usage is linked to the acquisition of Pro326 in the Env V3 crown that is near-universally conserved in SIVcpz and HIV-1. Furthermore, we found that expression of CXCR6 is enriched on CD4+ Tem cells in SMs, and is present on a population of cells distinct from that expressing CCR5. Together, these findings suggest that viral entry and cell tropism is CXCR6/CCR5-mediated in those SIV hosts, which differs from the CCR5-restricted entry pathway of SIVcpz and HIV-1, and that coreceptor tropism may have shifted with cross-species transfer and emergence of SIVcpz.

While it is well established that SIVs can use several human 7TMR coreceptors to enter cells *in vitro*, CCR5 was long considered the sole relevant SIV coreceptor *in vivo*. However SIVrcm, whose RCM host is frequently CCR5-null due to a common deletion allele, was recognized years ago to use CXCR6 and CCR2b, but not CCR5, *in vitro*, and to be CCR5-independent *in vivo* [36, 37]. More recently, we showed that both SIVsmm and SIVagmSab infections did not require CCR5 *in vivo* and/or *ex vivo* and efficiently used CXCR6 in addition to CCR5 [38–41]. Similar findings were also reported for SIVagmVer [42]. Here, we provide the first functional analysis of SIVmus Env. While MUS lymphocytes were not available to test primary cell infection using a CXCR6 blocking agent, as was done in our SM and AGM studies [40, 41], our results nevertheless identify another distinct SIV lineage that appears to share this entry pathway. These findings raise the possibility that CXCR6 tropism is a broadly shared feature of natural SIV infections and, furthermore, links this pattern directly to the HIV-1 ancestor lineage.

While SIVmus efficiently used species-matched CXCR6 for entry, SIVcpz could not. This pattern held for four diverse SIVcpz isolates representing both subspecies of chimpanzees infected in the wild, including the SIVcpz*Ptt* lineage that crossed into humans to found the HIV-1 pandemic [1]. SIVcpz was initially thought to be nonpathogenic in chimpanzees, but subsequent study of wild-living populations and captive primates revealed increased mortality and AIDS-like disease in infected apes [10, 61, 62]. While SIVsmm/SM and SIVagm/AGM infections are well-studied and typically nonprogressive (although a rare case of AIDS-like disease in a very old SIV+ SM has been reported [63]), the consequences of SIV infection in naturally-infected GSN/MUS/MON monkeys has not been investigated directly. Additionally, it is not possible to say definitively that coreceptor use by current-day members of this virus lineage accurately reflect the ancestor that contributed Env to the SIVcpz/HIV-1 lineage, although shared CXCR6 use among multiple naturally-infected monkey species suggests that this is plausible. Thus, a switch from dual-tropic CXCR6/CCR5 cell targeting in the SIVmus lineage to exclusive CCR5 use by SIVcpz may coincide with the transition to a more pathogenic infection. An intriguing parallel is seen in the nonpathogenic SIVsmm/SM versus pathogenic SIVmac/RM animal models, where SIVsmm enters SM lymphocytes via both CXCR6 and CCR5, while SIVmac that arose from SIVsmm uses only CCR5 to infect macaque CD4+ T cells [40, 42, 64]. Interestingly, in contrast to SIVcpz, where the lack of CXCR6 use is due to Env determinants, the restricted coreceptor use in SIVmac/RM is due to an Arg31 residue present in the rmCXCR6 N-terminus that renders it a poor coreceptor for SIVmac [43].

Differences in cell and tissue targeting between SIVsmm and SIVagm versus HIV-1 and SIVmac infections have been implicated in the distinct outcomes of infection [17, 18], raising the intriguing possibility that differential coreceptor use and expression may be responsible. HIV-1 and SIVmac infections are characterized by a greater loss of CD4+ T cell subsets, such as Tcm, Tscm, and Th17, compared to SIVsmm and SIVagm infections [17–21]. This is believed to contribute to the inability to maintain T cell homeostasis, as well as the loss of gut mucosal integrity, microbial translocation and chronic immune activation, which are all absent in SIVsmm and SIVagm infections. Additionally, infected humans and macaques exhibit extensive infection in lymph node follicles, with inflammation, fibrosis and structural disruption, features that are again absent in SIVsmm and SIVagm infections [22, 65–67]. Initial studies investigating SIV target cells identified low levels of CCR5 expression as a common feature of several natural host species [35], which may limit infection of certain critical CD4+ T cell subsets, such as Tcm and Tscm [17, 18]. Our observation that efficient CXCR6-mediated entry is a common feature of SIVsmm, SIVagm and now SIVmus infections suggests that use of this pathway may enable robust replication in the face of limited CCR5. Whether CXCR6 use by SIV strains from these multiple primate species was selected due to such a role, or arose as consequence of selection for other features to which it may be genetically linked, remains to be determined. Regardless, restricted CCR5 expression and use of CXCR6 together likely define which target cell subsets become infected and serve as a source of viremia in these natural SIV hosts.

Until now, it has not been possible to identify the cell subsets that express CXCR6 in SIV hosts due to lack of suitable reagents. Here, we report the generation of a new antibody that recognizes all primate CXCR6 molecules tested, and enabled interrogation of CXCR6 expression of SM CD4+ T cells. This revealed two important findings. First, in SMs, CXCR6 is enriched in Tem cells, and there is essentially no CXCR6 expression on naïve or central memory CD4+ cells. It is thus tempting to speculate that use of CXCR6, combined with highly restricted CCR5 expression in natural hosts, would direct SIV to cell types that are more easily replenished and whose infection and loss are less likely to disrupt immune homeostasis. The second finding is that in this species, CXCR6 and CCR5 are expressed on largely distinct CD4+ T cells populations in the blood, with little co-expression. This is in contrast to what has been reported in humans and mice, where CCR5 and CXCR6 are substantially coexpressed [68]. These data suggest that in SMs, CXCR6 is not simply an alternative entry route for SIVsmm into CCR5+ Tem cells, but rather targets a different CD4+ T cell population. Unfortunately, lymphocytes from MUSs or GSNs are not available to directly examine CXCR6 expression on CD4+ T cells in these species. It will also be important to determine CXCR6 expression on Th17 and T follicular helper cells in SMs, and in anatomical sites such as lymph nodes and the gut. Studies of humans and mice show that CXCR6 expression is restricted to extralymphoid sites [69, 70], and if true for monkeys naturally infected with SIV, could enable viral replication while avoiding lymphoid tissue infection that leads to inflammation, fibrosis and damage seen in pathogenic infections. Future studies also will be important to define the relationship between coreceptor expression and cell-associated viral DNA, and CXCR6 expression patterns in AGMs, which are known to downregulate CD4 expression to limit infection of memory CD4+ T cells [71] as well as CXCR6 expression in lymphoid tissue compartments across species.

Loss of CXCR6 tropism by SIVcpz is due in part to the acquisition of Pro326 in the Env V3 loop crown, which is highly conserved in SIVcpz and HIV-1. In contrast, no SIV isolate that has been experimentally confirmed to use CXCR6 encodes Pro at this position (such as SIVsmm and SIVagm, Fig 5B), nor does any natural host SIV that has been sequenced, including SIVdrl that infects drills (*Mandrillus leucophaeus*), SIVsyk that infects Sykes’ monkeys (*Cercopithecus albogularis*), and others [59]. Several rare HIV-1 isolates have been identified that can use CXCR6 *in vitro*, at least one of which encodes Trp instead of the usual Pro at this residue [33]. Since neither the P326A mutation nor introduction of the SIVmus V3 region conferred CXCR6 use on to SIVcpz, additional sequences beyond the V3 loop of Env are also required. This finding is consistent with studies showing that both V3 and additional sequences were necessary to confer CXCR6 use from an SIV to HIV-1 [72]. Studies of HIV-1 have shown that Pro326 regulates the molecular interactions between Env and CCR5 [73], suggesting that CXCR6/CCR5 tropic SIVs may interact with CCR5 in a manner distinct from CCR5-restricted viruses. A key question therefore is what prompted the acquisition of Pro326 and, consequently the loss of CXCR6 use, following the emergence of SIVcpz. Chimpanzees may have provided a more CCR5-rich environment in which CXCR6 use was no longer necessary, but since both Pro and Ala are compatible with CCR5 use, there likely are additional factors that favor Pro in SIVcpz. Other gp120 residues have been identified that differ between ape and monkey immunodeficiency viruses [74, 75] and further studies will be useful to determine whether they contribute to the dichotomous nature of CXCR6 use.

Beyond CXCR6, several other alternative coreceptors are used *in vitro* by various SIVs, particularly GPR15. However, SIVmus did not enter cells expressing species-matched GPR15. Previous studies identified Pro321 in the Env V3 loop as necessary for use of GPR15 for SIVmac [76]. While this residue is found in SIVsmm and SIVagm, which do use GPR15 *in vitro*, SIVmus1085 encodes Asn321, which may explain its lack of GPR15 use. Thus, CXCR6 appears to be the principal coreceptor, in addition to CCR5, of SIVmus.

A limitation of our study is that current-day SIVmus/gsn/mon reflect descendants of the virus that contributed Env to SIVcpz, and thus can only serve a surrogate for that ancient predecessor. Additionally, the phenotype of SIVmus/gsn/mon infection has not been studied directly, and surveys of bushmeat have revealed that MUS, GSN and MON monkeys are infected in the wild at much lower frequencies (0–7%) than SMs and AGMs (~50%), which might indicate that virus-host coadaptation in these species may not be the same as in SMs and AGMs [8, 50, 77]. Finally, primary cells from these animals were not available to test coreceptor entry pathways directly through blocking studies (as we have done with SM and AGM [40, 41]), or define CXCR6 expression patterns on their CD4 T cells. Thus, the intriguing association between targeting CXCR6/CCR5 versus CCR5-only CD4+ T cell subsets and pathogenic infection outcomes in the HIV-1/SIVcpz/SIVmus/gsn/mon lineage requires further study.

In summary, use of species-matched CXCR6 by yet another naturally occurring SIV, SIVmus, suggests a new paradigm for viral entry, cell tropism and targeting in SIV infection. In the natural host SM, CCR5 expression is exquisitely low, and CXCR6 appears to be confined to a unique subpopulation of differentiated CD4+ T cells that may be easily replenished and/or localized in such a manner that immune homeostasis and function are not disrupted, and this may also be true for other SIV infected primate species. It is thus possible that upon entry into a host with more abundant expression of CCR5, CXCR6 use was lost, resulting in altered cell tropism that may have contributed to an overall more pathogenic outcome.

## Materials and methods

### Cloning MUS and GSN coreceptors

Stored GSN and MUS gDNA was available from bushmeat samples previously obtained in Cameroon by the laboratory of M. Peeters [6, 49, 50]. Samples used in this study were MUS1085 [6] and MUS1246 [49] (both SIV+), and GSN1289 and GSN1365 (both SIV-) [50] and GSN42 (SIV+) [50]. Based on gDNA availability, MUS coreceptors were cloned from MUS1085 while GSN CCR5, CXCR6 and GPR15 were cloned from GSN1365 and CXCR4 was cloned from GSN1289.

The single-exon coreceptors CXCR6, CCR5 and GPR15 were amplified from MUS and GSN gDNA using primers and methods previously described for amplifying SM and AGM coreceptors [39, 41]. Briefly, primers that lie outside the coreceptors’ open reading frames and contain restriction sites were used to amplify MUS and GSN molecules using Phusion polymerase (New England Biolabs), and amplicons were ligated into pcDNA3.1+ (Invitrogen).

For CXCR4, which contains two exons (15 and 1044 bp respectively), each exon was amplified independently from gDNA using the following primers:

CXCR4 Exon 1 Fwd: 5’-**CACC**GGATCCGCCTGAGTGCTCCAGTAGCCACCGCATCTGG-3’

CXCR4 Exon 1 Rev: 5’-CACATGCAGCCACTGGAACGCTCT-3’

CXCR4 Exon 2 Fwd: 5’-TCACTATGGGAAAAGATGGGGAGGA-3’

CXCR4 Exon 2 Rev: 5’-GTCCCTCGAGACATCTGTGTTAGCTGGAGTGAAAACTTGAA-3’

(Sequences that permit TOPO Directional and/or restriction enzyme digest cloning are bolded or underlined, respectively.)

The amplicons were sequenced and a new forward primer designed to amplify the second exon that incorporated the short first exon in order to splice the two exons together. Because the MUS and GSN sequences were identical in this region, the same primer was used for both species: 5’-**CACC**ATGGAGGGGATCAGTATATACACTTCAGATAAC-3’ (exon 1 sequence is underlined and TOPO cloning sequence bolded). The CXCR4 splice product was amplified using Phusion polymerase and the product was inserted into pcDNA3.1/V5-His-TOPO vector using the pcDNA3.1 Directional TOPO Expression kit (Invitrogen).

Coreceptor clones were isolated, Sanger sequenced, and analyzed using the ClustalW algorithm with MacVector 15.5.0.

### MUS and GSN CD4

Because MUS and GSN cDNA was not available, we amplified the nine exons of CD4 from gDNA in four amplicons using primers flanking the exons, which were designed based on sequences shared by humans and rhesus macaques:

Exons 1–2 Fwd: 5’-CACCCAGCAAGGCCACAATGAAC-3’

Exons 1–2 Rev: 5’-TCAGACACCAAAGGCTTTCA-3’

Exons 3–4 Fwd: 5’-CCCAGCCAGGTAAATGGATA-3’

Exon 3–4 Rev: 5’-TCTCCACTCCTGACCTCCCA-3’

Exons 5–6 Fwd: 5’-GGAGAGGTAGGAAGGAACTGAAG-3’

Exons 5–6 Rev: 5’-GTCTCTGCCAACCACAGGAA-3’

Exons 7–9 Fwd: 5’-AAACCGATTCCCCAGCACT-3’

Exon 7–9 Rev: 5’-GGATCTGCTACATTCATCTGGT-3’

For samples with sufficient DNA (MUS1085, GSN42, GSN1289), all nine exons were amplified. For samples with limiting amounts of gDNA (MUS1246, GSN1365), only exons 1–4 (which include domain 1, encoding the gp120-interaction regions) were amplified. PCR was performed using Phusion polymerase and the product was run on an agarose gel. Amplicons of the proper size were purified and sequenced using a MiSeq sequencer (Illumina) and analyzed using Geneious 7 software. For MUS CD4 one allele was selected, and for GSN CD4 a consensus sequence was generated, and the full coding sequence for each species was then synthesized and cloned into an expression vector pcDNA3.1+ (GenScript).

### Chimpanzee coreceptors and CD4

CD4-depleted PBMCs that were non-viably frozen, left over from previous studies from the laboratory of B. Hahn, were available from one *Pan troglodytes verus* animal. Cells were thawed and DNA and RNA were isolated using the QiaAmp Blood Mini Kit (Qiagen) and the RNeasy kit (Qiagen), respectively.

Chimpanzee CXCR6, CCR5 and GPR15 were cloned from gDNA using the PCR primers and method described above for MUS and GSN. To clone chimpanzee CXCR4, cDNA was synthesized from RNA using a gene-specific primer for CXCR4 and the Superscript III kit (Invitrogen) followed by PCR amplification using primers outside the open reading frame and protocols as previously described for cloning SM CXCR4 [39].

Cloning of chimpanzee CD4 has been previously described and an allele found across chimpanzee subspecies was used here [54].

### SIVmus *env* cloning

SIVmus *env* was cloned from bushmeat gDNA that was previously determined to be SIV-positive by M. Peeters [6, 49]. Primers to amplify SIVmus Envs were designed based on available sequence data for SIV from MUS1085 (accession number AY340700) and MUS1246 (accession number EF070329). PCR was performed in two rounds using Expand HF PCR System (Roche), following the manufacturer’s protocol and an annealing temperature of 55°C. Round one (35 cycles) contained outer primers and at least 100ng of gDNA. Round two (45 cycles) employed 2uL of the round 1 product as input, and was done in duplicate employing two different sets of inner primer pairs. Primer sequences are below: set 1 is the outer set, while sets 2 and 3 are the two inner sets (TOPO cloning sequences on inner primers are bolded). For both MUS 1085 and MUS 1246, both inner primer sets yielded *env* amplicons.

MusEnv1085Fwd1: 5’-GTGGAATTTGGAATGAGGTAACGG-3

MusEnv1085Rev1: 5’-TGGTCTAGGAGGTATTGGTCATCT-3

MusEnv1085Fwd2: 5’-**CACC**TGCTTTTCATTGCGTACTCTGTTT-3

MusEnv1085Rev2: 5’-TATCACAGGTCTTTTACTGGCTCC-3

MusEnv1085Fwd3: 5’-**CACC**ACCTCCTTTGAGTCCTTCTAGGTA-3

MusEnv1085Rev3: 5’-GAAATGCGACATATCCACCATCAG-3

MusEnv1246Fwd1: 5’-ATCCACAATGGTCTGTAGATCAGG-3

MusEnv1246Rev1: 5’-CAAAAGGAGAAGACCAGGAACTCT-3

MusEnv1246Fwd2: 5’-**CACC**ATGTTGCGCGTTTCACTGTATATT-3

MusEnv1246Rev2: 5’-AGTTTTTGCAGTCTGTCTATGCAC-3

MusEnv1246Fwd3: 5’-**CACC**TCTAATTCCATGCCAAATGCTGAC-3

MusEnv1246Rev3: 5’-GCACACAAACATTCCTTCTAGTCC-3

PCR amplicons were confirmed by agarose gel, column purified and ligated into pcDNA3.1/V5-His-TOPO (Invitrogen). Ligation products were transformed into Stbl2 cells, cultured at 30°C to prevent insert deletion, and colonies screened for inserts by colony PCR. Plasmids with properly sized inserts were screened for functionality by generating pseudotyped viruses with backbone pNL4-3Luc E-R+, and testing on human embryonic kidney 293T (293T) cells transfected to express CD4 and coreceptor as previously described [41]. This yielded five distinct functional Envs from MUS 1085, three of which (1–54, 4–1 and 4–12) were used in this study. From MUS 1246, this yielded five distinct Envs, but all functioned very poorly or not at all and thus were not analyzed.

### SIVcpz isolates and Envs

Infectious molecular clones (IMCs) have been previously reported for SIVcpz*Ptt* strains MB897, EK505 and MT145 [78], and SIVcpz*Pts* strain BF1167 [44]. Infectious SIVcpz virus stocks were generated by transfecting IMC plasmids into 293T cells. Cells were washed and media changed 24 hours post transfection, and supernatant was harvested 48 hours later. Codon-optimized *env* genes from strains EK505 and MT145, which were used for SIVcpz Env pseudotyped viruses, were generated as previously reported [79].

### SIVmus and SIVcpz *env* mutagenesis

Point mutations were introduced into SIVmus1085 1–54 and SIVcpz*Ptt* MT145 *envs* with the Quickchange II XL Site-Directed Mutagenesis Kit (Agilent) using primers and protocols per manufacturer’s instructions. To introduce the V3 region of SIVmus into SIVcpz (SIVcpz-musV3), the SIVcpzPtt MT145 *env* plasmid was linearized by performing PCR using outward primers that flanked the V3 loop, such that the entire plasmid excluding the V3 loop was amplified. The V3 region of SIVmus1085 1–54 was amplified using primers that contained ~20 bases of homology to SIVcpz MT145 immediately flanking the V3 loop. The two amplicons were gel purified and assembled using the NEBuilder HiFi DNA Assembly Cloning Kit (New England Biolabs). Mutations and exchanges were confirmed by sequencing.

### Analysis of coreceptor function using IMC-derived SIVcpz

Replication competent SIVcpz were tested in CF2th-Luc cells, which contain a Tat-responsive luciferase reporter gene and were originally generated in the laboratory of J. Sodroski [55]. Cells were plated at 5x10^5^ cells/well in 6-well plates and transfected with CD4 and coreceptor plasmids (1 ug each) using Fugene 6. Cells were washed and replated in 96-well plates 24h later. One day later cells were infected with 50uL of IMC-derived virus stock. Cells were lysed 48 h later and luciferase content measured by adding luciferase substrate and reading relative light units (RLU) on a Luminoskan Ascent instrument.

### Analysis of coreceptor function using luciferase reporter pseudotyped virus

Pseudotyped viruses were generated as previously described [39] by transfecting 293T cells with plasmid containing SIVcpz, SIVmus, SIVgsn or mutant *envs* along with the luciferase reporter backbone pNL4-3LucE-R+ using Fugene 6 reagent. Cells were washed one day later, and supernatant was harvested 48 hours after that. Virus was quantified using a p24 ELISA (Perkin-Elmer).

Pseudotype infection of 293T cells expressing CD4 and coreceptor were performed as previously described [41]. Briefly, cells were transfected with species-specific CD4 and coreceptor plasmids, and replated 24h later into 96-well plates. The following day, cells were infected by spinoculation (1200g for 2 hours) with DNAse-treated pseudotyped viruses, incubated for 72 hours, and then luciferase content measured in cell lysates. Pseudotyped viruses carrying the VSV glycoprotein served as a positive control. To confirm functional CD4 and coreceptor expression, transfected cells were infected in parallel with pseudotyped viruses carrying Env from previously described SIVsmm (FTv3.1 and FJv2.1) that use a broad repertoire of coreceptors [39].

### Generation of anti-primate CXCR6 monoclonal antibody 20D8

To generate a CXCR6-expressing cell to use as an immunogen, a plasmid was made with GFP fused to the C-terminus of SM CXCR6 (smCXCR6-GFP). Appropriate conformation of the coreceptor was confirmed by the ability of smCXCR6-GFP to support SIVsmm entry. The smCXCR6-GFP construct was then inserted into the lentiviral vector pELNS. Murine B78H1 cells (provided by Gary Cohen, University of Pennsylvania) were then transduced with smCXCR6-GFP and a uniform population of GFP-high cells was selected by flow cytometry and used for immunization.

Balb/c mice were first immunized by a series of three hydrodynamic tail vein DNA injections with an expression plasmid containing native smCXCR6 (15 ug plasmid per injection) given one or two weeks apart. Five weeks after the last DNA immunization, mice began a series of 5 intraperitoneal injections, given every 2 weeks, of smCXCR6-GFP high-expressing B78H1 cells that had been sublethally irradiated (10 x 10^6^ cells per injections 1–3, 50 x 10^6^ cells per injections 4 and 5). One week after the final injection, spleens were harvested and hybridomas generated.

To identify an antibody that might have reactivity against CXCR6, hybridoma supernatants were screened for reactivity with smCXCR6 using a cell-based ELISA (cELISA; modified from [80]). 293T cells were transfected with plasmids containing smCXCR6 or empty vector, then seeded 48 hours later into 96 well plates (10^5^ cells/well) in PBS with 3%BSA. After 30 min at room temperature, plates were spun, buffer aspirated, and undiluted hybridoma supernatant added and incubated for 1 hour at 4°C. Cells were washed twice, incubated for 1 hour at room temperature in a 1:100 dilution of goat anti-mouse horseradish peroxidase (HRP) antibody (Cell Signaling Technology) in PBS with 3%BSA, washed again, then fixed with 3% paraformaldehyde. Cells were then washed with sodium citrate buffer (20mM pH 4.5). Citrate buffer was removed and HRP substrate (Rockland) added and allowed to develop for 15 minutes. Approximately 1400 hybridomas were screened, and one was identified (20D8) that was reactive by cELISA with smCXCR6 but not with empty vector-transfected control cells. Antibody 20D8 was then subject to IgG purification using standard methods. Of note, the same protocol was carried out in parallel using rmCXCR6 as the immunogen, but 20D8 from the smCXCR6 protocol yielded the best antibody.

Monoclonal antibody 20D8 was tested for cross-reactivity with other species’ CXCR6 and for specificity using other 7TMRs by flow cytometry. 293T cells were transfected with plasmids expressing CXCR6 of human, chimpanzee, MUS, GSN, RM, SM, sabaeus AGM, or vervet AGM origin, as well as SM CCR5, CXCR4, APJ or GPR15, or empty vector. Cells were detached 48 hours later and stained with unconjugated 20D8 followed by APC-conjugated (Fig 6) or PerCP-Cy5.5-conjugated (S3 Fig) goat anti-mouse IgG (Poly4053, BioLegend), the secondary antibody alone, or an anti-human CXCR6 AF647 antibody (K041E5, BioLegend). Cells were washed and analyzed by flow cytometry.

To further establish the CXCR6 specificity of antibody 20D8, human PBMC from two healthy donors were stained with 20D8 and sorted into positive and negative populations on a FACSAria II (Becton-Dickinson). RNA was extracted, converted into cDNA using random hexamers, and then subjected to qPCR for CXCR6 and GAPDH expression. In parallel, GHOST-CD4 and GHOST-CXCR6 (also known as GHOST-Bonzo) cells were analyzed, which are HOS cells stably transfected to express high levels of CD4, or both CD4 and CXCR6, respectively [81]. PCR was carried out with Sybr Green using primers: CXCR6-F: 5'-GGTTCTTCTTGCCACTGCTC-3'; CXCR6-R: 5'-CATGAGGTTGAAGGGCATCT-3'; and GAPDH-F: 5'-CTGTTCGACAGTCAGCCGCATC 3'; GAPDH-R: 5'-GCGCCCAATACGACCAAATCCG-3'. Expression of CXCR6 relative to GAPDH in the 20D8 positive sorted PBMC relative to the 20D8 negative population, or GHOST-CXCR6 relative to GHOST-CD4, was calculated using the ΔΔCt method [82].

### CXCR6 expression on sooty mangabey PBMC

PBMC from six SMs and six RMs were previously isolated and stored in the laboratories of G. Silvestri and M. Paiardini. Five SM were homozygous wild type for CCR5, and one was heterozygous for the CCR5Δ2 mutation [38]. Cryopreserved cells were thawed and rested overnight in RPMI supplemented with 10% FBS, 1% L-glutamine and 1% pen/strep prior to staining. Cells were first stained with the anti-CXCR6 clone 20D8, followed by a goat anti-mouse IgG AF488-conjugated secondary antibody (Poly4053, BioLegend). Cells were washed and then stained with Aqua Live/Dead Dye (ThermoFisher) and the remaining surface antibodies: anti-CD3 APC-Cy7 (clone SP34-2, BD Pharmingen), anti-CD4 PECy5.5 (clone S3.5, Invitrogen), anti-CD8 BV570 (clone RPA-T8, BioLegend), anti-CCR7 BV711 (clone G043H7, BioLegend), anti-CD95 PECY5 (clone DX2, BD Pharmingen) anti-CD28 ECD (clone CD28.2, Beckman Coulter), anti-CCR5 PE (clone 3A9, BD Pharmingen), anti-CD45RA PE Cy7 (clone 5H9, BD Pharmingen), anti-CD20 BV650 (clone 2H7, BD Horizon), anti-CD16 BV650 (clone 3G8, BioLegend), anti-CD14 BV605 (M5E2, BioLegend). Cells were stained in parallel with the entire panel but with unconjugated 20D8 antibody omitted. The gate for CXCR6 expression was then drawn on the cells stained with the secondary antibody only, and the percent of cells positive for CXCR6 was calculated by subtracting the percent of AF488 positive cells in the secondary only condition from the anti-CXCR6 20D8 plus secondary condition. Samples were run on an LSRII (BD) and data analyzed using FlowJo software (v. 9.9).

For mitogen stimulation experiments, thawed and rested PBMC from each SM were divided and either stained for baseline coreceptor expression, or stimulated with 5ug/mL concanavalin A (Sigma) and maintained in media supplemented with 100IU/mL IL-2. Aliquots of stimulated cells were removed on days 5, 7 and 9 post stimulation for analysis. These resting and stimulated cells were stained for viability, CD3, CD4, CD8, CCR5 and CXCR6, and analyzed as described above.

### Ethics statement

Mouse immunization and spleen harvesting was done by Cocalico Biologicals (Stevens, PA). The Cocalico Biologicals, Inc. Animal Care and Use Committee reviewed and approved the animal care and use protocols used for mouse studies to generate antibody 20D8 (2014–0556 and 2014–0558). These animal care and use protocols adhered to regulations and guidelines as outlined in the Animal Welfare Act (USDA) and the guidelines set forth in the Guide for the Care and Use of Laboratory Animals: Eighth Edition (OLAW).

### Sequences

The following sequences have been deposited with GenBank: musCCR5, musCXCR6, musGPR15, musCXCR4, gsnCCR5, gsnCXCR6, gsnGPR15, gsnCXCR4, cpzCCR5, cpzCXCR6, cpzGPR15, cpzCXCR4, cpzAPJ, cpzCCR2b, cpzCCR4, cpzCCR3, cpzCCR8, and cpzGPR1 (MG267399-MG267416); SIVmus1085 and SIVmus1246 *envs* (MG450752-MG450761).

## Supporting information

S1 Fig SIVcpz does not use cpzAPJ, CCR2b, CCR3, CCR4, CCR8 or GPR1 for entry. Top: CF2thLuc cells that contain a Tat-driven luciferase reporter were transfected with expression plasmids containing chimpanzee CD4 and coreceptor as indicated or empty vector (No CoR). 48 hours later cells were infected with one of four diverse SIVcpz isolates derived from infectious molecular clones. Entry was quantified 48 hours later by lysing cells and measuring luciferase content by relative light units (RLU). Bottom: As a positive control for coreceptor function, 293T cells were transfected with cpzCD4 and CPZ coreceptors and infected with luciferase reporter pseudotypes carrying a promiscuous SIVsmm Env that can use a wide repertoire of coreceptors for entry. Infections were carried out in triplicate and error bars represent one standard deviation. These coreceptors were tested in parallel to those displayed in Fig 1, and the same data for No CoR and cpzCCR5 are displayed for comparison.
(TIF)

S2 Fig Coreceptor use patterns of SIVcpz EK505 and SIVmus1085 4–12 also suggest that failure to use cpzCXCR6 is Env determined. 293T cells were transfected with expression plasmids containing CD4 and coreceptor. The species origin of the CD4 and coreceptor are indicated below the graph (C, chimpanzee; M, mustached monkey;–, empty vector). 48 hours post transfection, cells were infected with luciferase reporter pseudotypes carrying the SIVcpz EK505 Env (A) or the SIVmus1085 4–12 Env (B). Entry was quantified 72 hours later by lysing cells and measuring luciferase content by relative light units (RLU). Infections were carried out in triplicate and error bars represent one standard deviation.
(TIF)

S3 Fig Antibody 20D8 detects CXCR6 but does not cross-react with other 7TMRs of sooty mangabey origin, and selects for PBMC enriched in CXCR6 RNA. A) 293T cells were transfected with expression plasmid containing CXCR6, CCR5, CXCR4, APJ or GPR15 of sooty mangabey origin or with empty vector. 48 hours later, cells were stained with anti-CXCR6 antibody 20D8 followed by a goat anti-mouse secondary. CXCR6-expressing cells were also stained with the secondary antibody alone. B) Human PBMCs from two donors were sorted into 20D8 positive and negative populations, and subjected to qPCR for expression of CXCR6 RNA relative to GAPDH RNA (left panel). Expression of CXCR6 RNA in 20D8-positive cells is shown relative to sort-negative cells. In parallel, CXCR6 expression was determined in GHOST-CXCR6 cells, which are HOS cells stably transfected to express high level CXCR6, relative to GHOST-CD4 cells (right panel).
(TIF)

S4 Fig CXCR6 expression on rhesus macaque CD4+ T cells. A) Expression on RM resting CD4+ T cells of CXCR6 (x-axis) and CCR5 (y-axis). Numbers in the quadrant are the percent of CD4+ T cells expressing the respective combination of coreceptors. B) Resting PBMC from 6 RM were stained using antibodies to define CXCR6 expression of CD4+ memory subsets: naive (Tn: CD45RA+/ CCR7+/ CD28+/ CD95-), central memory (Tcm: CD45RA-/ CCR7+) and effector memory (Tem: CD45RA-/ CCR7-). Data show individual percentages, along with mean and standard deviation. Each symbol represents cells from a different individual RM.
(TIF)

S5 Fig Regulation of CXCR6 and CCR5 on SM CD4+ T cells upon stimulation. SM PBMC from six animals were stimulated with concanavalin A and IL-2. Staining for expression of CD4, CXCR6 and CCR5 was done prior to stimulation, and at days 5, 7 and 9 post-stimulation. Data show individual percentages, along with mean and standard deviation. Each symbol represents cells from a different individual SM at each time point.
(TIF)
