# Supplementary figures and images for: Loss of CXCR6 coreceptor usage characterizes pathogenic lentiviruses
Source: PLoS Pathog. 2018 Apr 16;14(4):e1007003. doi: 10.1371/journal.ppat.1007003 (PMC5919676; doi:10.1371/journal.ppat.1007003)

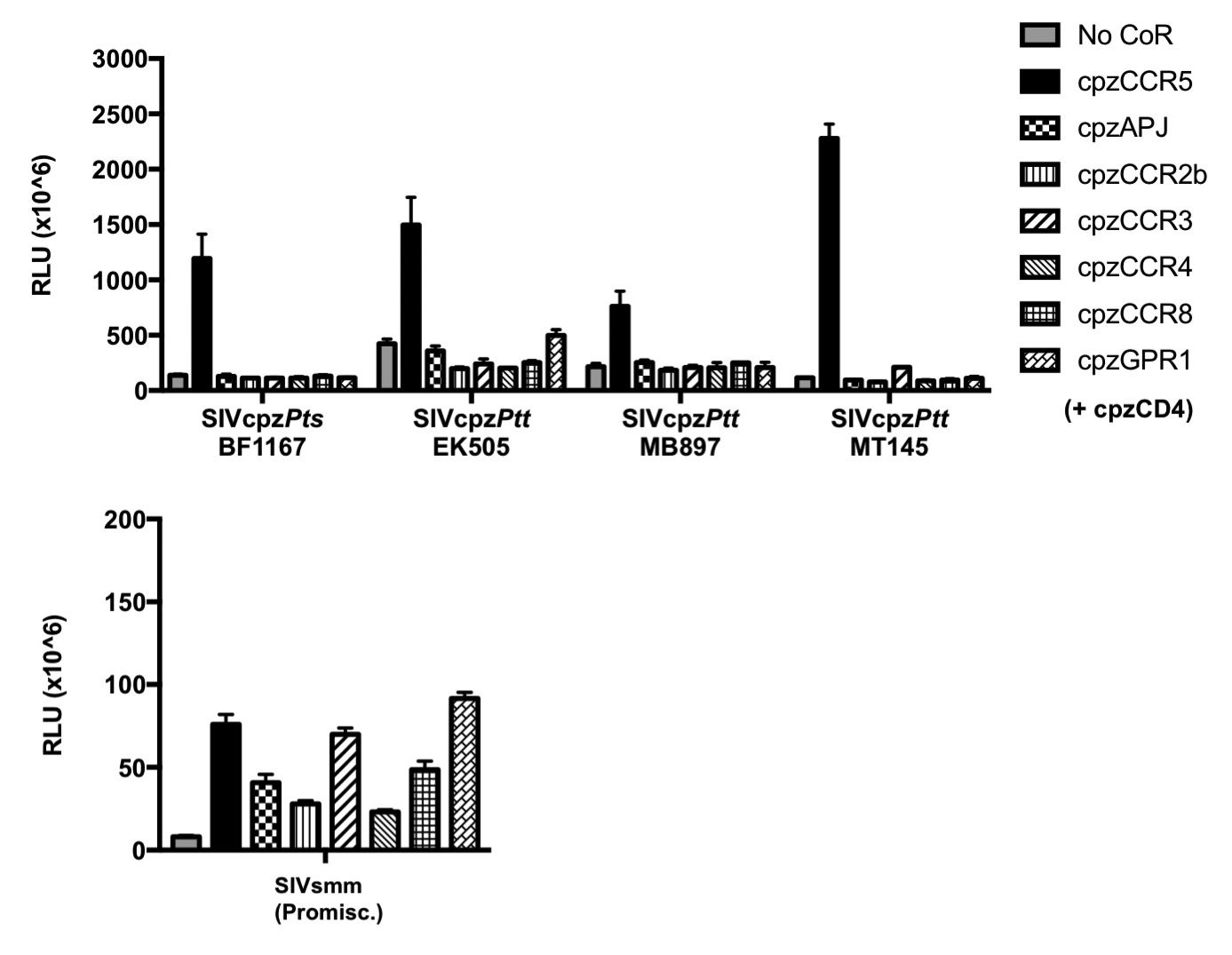

Supplement: S1 Fig — Top: CF2thLuc cells that contain a Tat-driven luciferase reporter were transfected with expression plasmids containing chimpanzee CD4 and coreceptor as indicated or empty vector (No CoR). 48 hours later cells were infected with one of four diverse SIVcpz isolates derived from infectious molecular clones. Entry was quantified 48 hours later by lysing cells and measuring luciferase content by relative light units (RLU). Bottom: As a positive control for coreceptor function, 293T cells were transfected with cpzCD4 and CPZ coreceptors and infected with luciferase reporter pseudotypes carrying a promiscuous SIVsmm Env that can use a wide repertoire of coreceptors for entry. Infections were carried out in triplicate and error bars represent one standard deviation. These coreceptors were tested in parallel to those displayed in Fig 1, and the same data for No CoR and cpzCCR5 are displayed for comparison. (TIF) [file ppat.1007003.s001.tif]

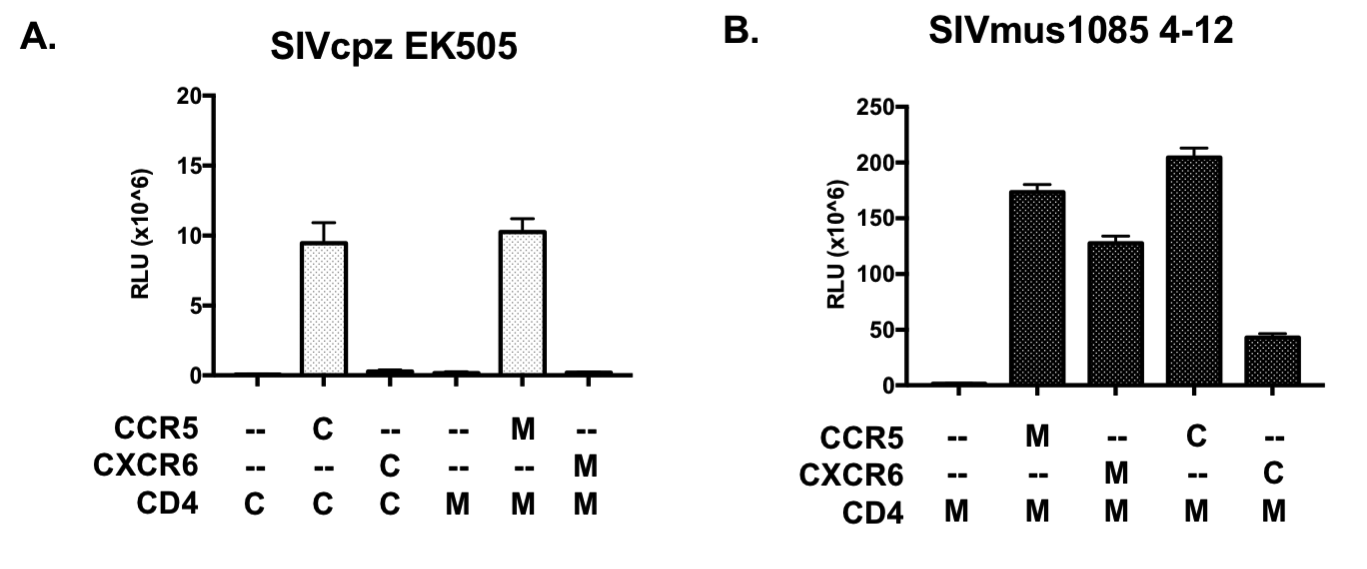

Supplement: S2 Fig — 293T cells were transfected with expression plasmids containing CD4 and coreceptor. The species origin of the CD4 and coreceptor are indicated below the graph (C, chimpanzee; M, mustached monkey;–, empty vector). 48 hours post transfection, cells were infected with luciferase reporter pseudotypes carrying the SIVcpz EK505 Env (A) or the SIVmus1085 4–12 Env (B). Entry was quantified 72 hours later by lysing cells and measuring luciferase content by relative light units (RLU). Infections were carried out in triplicate and error bars represent one standard deviation. (TIF) [file ppat.1007003.s002.tif]

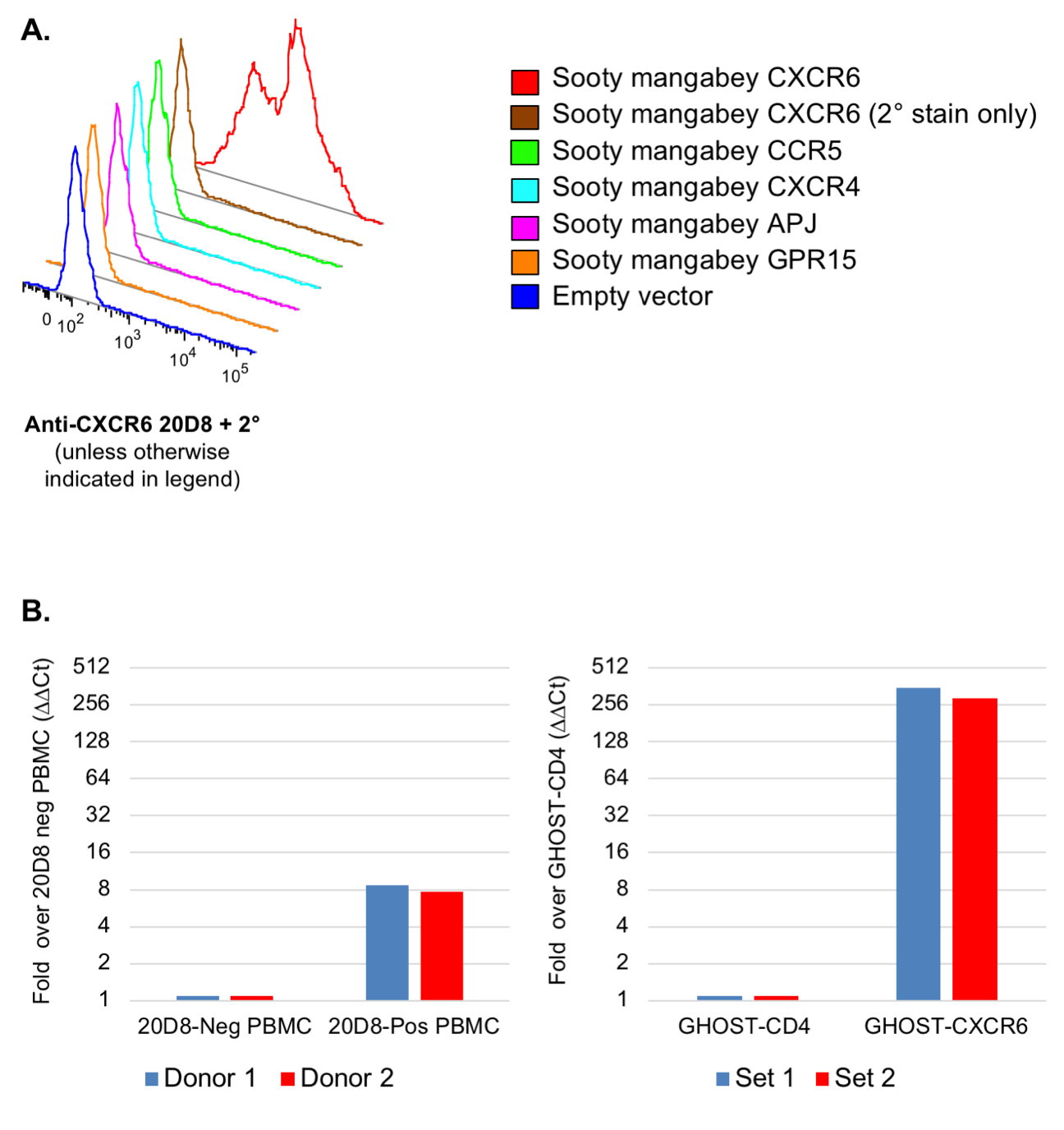

Supplement: S3 Fig — A) 293T cells were transfected with expression plasmid containing CXCR6, CCR5, CXCR4, APJ or GPR15 of sooty mangabey origin or with empty vector. 48 hours later, cells were stained with anti-CXCR6 antibody 20D8 followed by a goat anti-mouse secondary. CXCR6-expressing cells were also stained with the secondary antibody alone. B) Human PBMCs from two donors were sorted into 20D8 positive and negative populations, and subjected to qPCR for expression of CXCR6 RNA relative to GAPDH RNA (left panel). Expression of CXCR6 RNA in 20D8-positive cells is shown relative to sort-negative cells. In parallel, CXCR6 expression was determined in GHOST-CXCR6 cells, which are HOS cells stably transfected to express high level CXCR6, relative to GHOST-CD4 cells (right panel). (TIF) [file ppat.1007003.s003.tif]

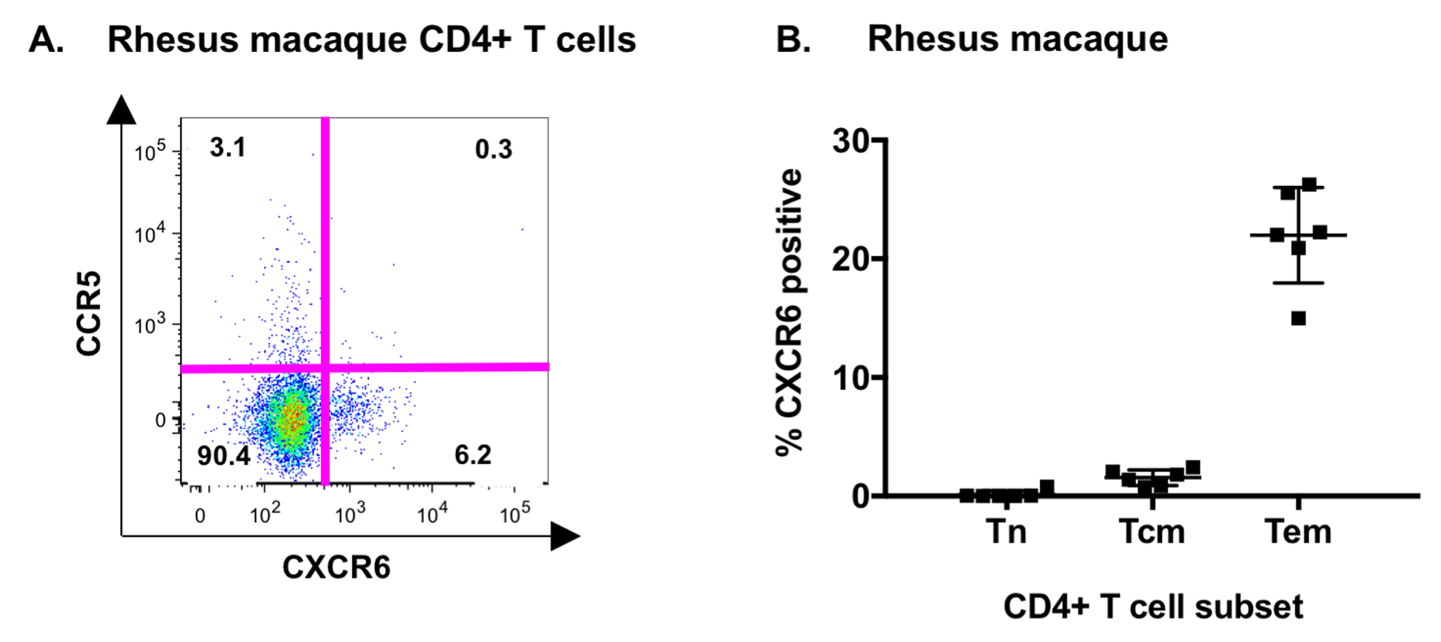

Supplement: S4 Fig — A) Expression on RM resting CD4+ T cells of CXCR6 (x-axis) and CCR5 (y-axis). Numbers in the quadrant are the percent of CD4+ T cells expressing the respective combination of coreceptors. B) Resting PBMC from 6 RM were stained using antibodies to define CXCR6 expression of CD4+ memory subsets: naive (Tn: CD45RA+/ CCR7+/ CD28+/ CD95-), central memory (Tcm: CD45RA-/ CCR7+) and effector memory (Tem: CD45RA-/ CCR7-). Data show individual percentages, along with mean and standard deviation. Each symbol represents cells from a different individual RM. (TIF) [file ppat.1007003.s004.tif]

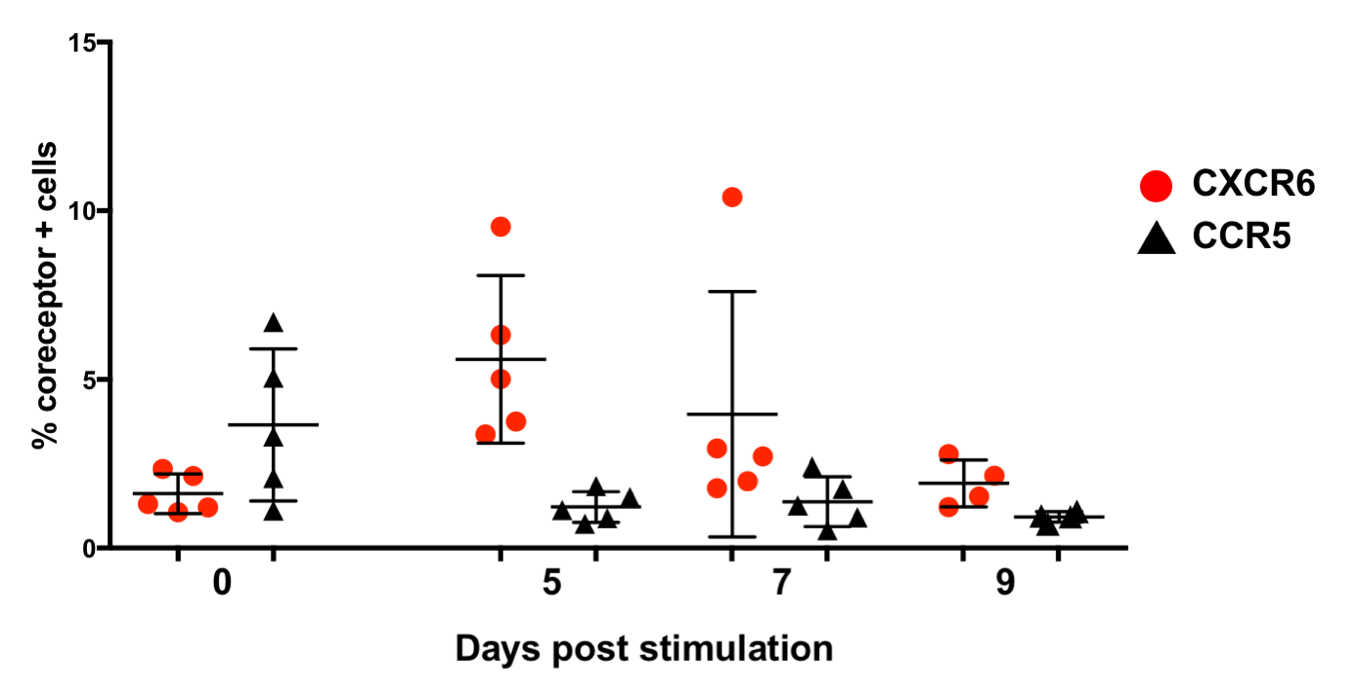

Supplement: S5 Fig — SM PBMC from six animals were stimulated with concanavalin A and IL-2. Staining for expression of CD4, CXCR6 and CCR5 was done prior to stimulation, and at days 5, 7 and 9 post-stimulation. Data show individual percentages, along with mean and standard deviation. Each symbol represents cells from a different individual SM at each time point. (TIF) [file ppat.1007003.s005.tif]
